# Genetic dissection of grain architecture-related traits in a winter wheat population

**DOI:** 10.1186/s12870-021-03183-3

**Published:** 2021-09-10

**Authors:** Matías Schierenbeck, Ahmad M. Alqudah, Ulrike Lohwasser, Rasha A. Tarawneh, María Rosa Simón, Andreas Börner

**Affiliations:** 1 Genebank Department, Leibniz Institute of Plant Genetics and Crop Plant Research (IPK), OT Gatersleben, Corrensstr 3, D-06466 Seeland, Germany; 2 Cereals, Faculty of Agricultural Sciences and Forestry, National University of La Plata, La Plata, Argentina; 3 CONICET CCT La Plata. La Plata, Buenos Aires, Argentina; 4 Department of Agroecology, Aarhus University at Flakkebjerg, Forsøgsvej 1, 4200 Slagelse, Denmark; 5 CICBA. La Plata, Buenos Aires, Argentina

**Keywords:** Thousand kernel weight, Winter wheat, GWAS, Grain architecture, Candidate genes

## Abstract

**Background:**

The future productivity of wheat (*T. aestivum* L.) as the most grown crop worldwide is of utmost importance for global food security. Thousand kernel weight (TKW) in wheat is closely associated with grain architecture-related traits, e.g. kernel length (KL), kernel width (KW), kernel area (KA), kernel diameter ratio (KDR), and factor form density (FFD). Discovering the genetic architecture of natural variation in these traits, identifying QTL and candidate genes are the main aims of this study. Therefore, grain architecture-related traits in 261 worldwide winter accessions over three field-year experiments were evaluated.

**Results:**

Genome-wide association analysis using 90K SNP array in FarmCPU model revealed several interesting genomic regions including 17 significant SNPs passing false discovery rate threshold and strongly associated with the studied traits. Four of associated SNPs were physically located inside candidate genes within LD interval e.g. BobWhite_c5872_589 (602,710,399 bp) found to be inside *TraesCS6A01G383800* (602,699,767–602,711,726 bp). Further analysis reveals the four novel candidate genes potentially involved in more than one grain architecture-related traits with a pleiotropic effects e.g. *TraesCS6A01G383800* gene on 6A encoding oxidoreductase activity was associated with TKW and KA. The allelic variation at the associated SNPs showed significant differences betweeen the accessions carying the wild and mutated alleles e.g. accessions carying C allele of BobWhite_c5872_589, *TraesCS6A01G383800* had significantly higher TKW than the accessions carying T allele. Interestingly, these genes were highly expressed in the grain-tissues, demonstrating their pivotal role in controlling the grain architecture.

**Conclusions:**

These results are valuable for identifying regions associated with kernel weight and dimensions and potentially help breeders in improving kernel weight and architecture-related traits in order to increase wheat yield potential and end-use quality.

**Supplementary Information:**

The online version contains supplementary material available at 10.1186/s12870-021-03183-3.

## Background

Meeting the globally increasing demand for wheat (*T. aestivum* L.), the main source of protein and calories in human food is a major aim to ensure global food security. By 2050, the world population will reach 9800 billion and the annual rate of food demand will reach 1.6% surpassing the current annual genetic gains of this crop [1].

The increase in grain yield had been based mainly on the increase in the number of grains per area [2, 3], whereas some other traits as thousand-grain weight (TKW) remained unchanged [4]. However, increasing grain yield and its potential could be reached through improving grain architecture-related-traits which helps in boosting TKW [5, 6].

Although the trade-off between the grain number and TKW is well known [7–9], no differences in TKW have been found in some genotypes with a high spikelet number [10] indicating that a high TKW can be achieved keeping the grain number unmodified and potentially increasing grain yield. This suggests that selection for heavier grains could be highly effective for improving wheat yields [11]. Therefore, understanding the genetic basis of grain architecture related-traits is important to accelerate the genetic gain of wheat grain yield. Nevertheless, TKW and kernel size are complex genetic traits controlled by multiple loci/genes with the influnce of environmental cues and genotype × environment (G × E) interactions [12, 13].

Kernel weight contributes about 20% of the genetic variation in grain yield. Besides that, kernel weight is a stable, heritable character, thus suitable to select in segregating generations in plant breeding [14]. Furthermore, TKW was also reported to increase seedling vigor and germination [15]. A higher TKW also generates a higher flour yield [16, 17]. In durum wheat, TKW, kernel volume and test weight are also associated with semolina yield [18].

Grain architecture-related traits can also be associated with milling and processing wheat quality [19, 20]. A close phenotypic association between TKW with other kernel size traits such as kernel length (KL), kernel width (KW), and kernel diameter ratio (KDR) in bread wheat has been recognized [15] and those traits have been reported as modifying milling quality [21]. It has been indicated that grain characteristics are important attributes for determining the market value of wheat grain since they influence the specific weight and milling performance. Moreover, Evers et al. [22] reported that large and spherical grains optimize grain morphology increasing milling efficiency. For their part, Millar et al. [23] described that kernel size was associated with various characteristics of flour, such as protein content and hydrolytic enzymes activity that are closely related to baking quality and end-use suitability whereas Morgan et al. [24] reported the association of kernel size with flour-water dough quality. Despite this, the phenotypic and genetic variation of grain morphology is surprisingly understudied compared to other grain yield components, mainly due to the difficulty in quantifying this trait [25–28].

Exploration of crop genetic resources is useful to recognize sources of variation for agronomic and physiological traits and discovery of new alleles for improving yield potential, their components as well as grain quality traits [11, 29, 30]. Moreover, quantitative trait loci (QTL) mapping is a key approach to understand the genetic architecture of kernel traits. This tool has been implemented in several crops and generates important progress in identifying major QTL and isolating underlying genes for grain weight and size in rice, maize, and barley [31–33]. For bread wheat, several QTL associated with kernel morphology have been reported in recombinant inbred lines (RIL) populations such as *qTKW-1A.1*, *qTKW-2D.4* and *QTkw.ncl-5B.2* on chromosomes 1A, 2D and 5B, respectively [20, 21, 32, 34, 35], doubled-haploid (DH) populations like *Xgwm234*, *XwPt0052* and *XwPt9824* on chromosomes 5B, 6B and 7A, respectively [15, 21, 36–38] and F_2_ populations containing synthetic hexaploid wheat lines such as *Xcfd282-Xbarc62, Xhbe341-Xbarc225* and *Xhbd166-Xcfd81* on chromosomes 1D, 4D and 5D, respectively [39]. Genome-wide association scan (GWAS) has been used to detect several genomic regions and candidate genes underlying the natural variation of TKW and other grain yield components [9, 11, 14, 25, 40–43]. Up to now, QTL associated with grain architecture traits in winter wheat have been reported (e.g., *AX_111147652* on 1B; *AX_110046841* on 4A; *AX_110713957* on 4B; AX*_110958315* on 5A; *IWB50649* on 5B) but new efforts are required exploring different environments and germplasm from different origins in order to enhance grain yield and quality.

In addition, most of what is known about genetic control of TKW had been carried out using bi-parental populations which present some limitations [34, 38, 44]. Several candidate genes have been associated with TKW and related traits QTLs [14, 41, 42]. For example, a pseudo-response regulator (*Ppd-A1*) at 2A (*TRITD2Av1G019250)*, *TRITD4Bv1G171270* at 4B encoding a Big Grain 1 protein, and two other candidate genes at 6B (*TRITD6Bv1G005370* and *TRITD6Bv1G005450*) encoding an acid β-fructofuranosidase have been detected within QTL for KL and TKW in recombinant inbred lines (RILs) population [45]. Interestingly, QTL for KA with candidate genes involved in auxin were detected, for instance, *TRITD1Bv1G118820*, *TRITD2Av1G189400* and *TRITD7Bv1G173200* encoding auxin response while *TRITD4Bv1G175480* involved in auxin signaling and *TRITD3Av1G012070*, encoding for a Flavin-containing monooxygenase as an auxin biosynthesis. Moreover, candidate genes encoding for cytochrome P450 were found in most of the TKW QTL [45].

Other candidate genes as *TraesCS4A02G229100* which is an auxin-regulated gene involved in organ size and *TraesCS4A02G2* corresponding to a polygalacturonase involved in carbohydrate metabolic process increasing kernel size and TKW [43] were found on chromosome 4AL. However, associations varied according to the environments and genotypes, indicating that new studies in different environments and germplasm should be carried out.

Therefore, to gain deeper insights into the genetic basis of grain architecture-related-traits that could be of interest for the future improvement of grain yield and quality, a GWAS was undertaken in a diverse winter wheat panel of 261 genotypes tested during 3 years. For this purpose, 17,093 SNPs markers with recently known physical position were used to detect the most significant and effective SNP and for the identification of candidate genes underlying the studied traits. Our results showed 17 highly associated SNPs across 9 chromosomes of which four multi-traits associated SNPs were reported on chromosomes 1B, 2A, 5B, and 6A. These SNPs were located within the physical positions of candidate genes which are potentially associated with grain architecture-related traits and potentially involved in improving grain weight and size.

## Results

### Population structure and SNP coverage

The markers were distributed within the whole genome. The highest coverage of markers (51.5%) was on genome B with 8809 SNPs, genome A was covered by 38.6% of the whole markers with 6595 SNPs, while D genome presented the lowest coverage, 9.9% (1689 SNPs). The homoeologous group 1 chromosome had the highest number of SNPs (17.96%), while the chromosomes of group 4 presented only 7.02%. Chromosome 5B harboured the highest number of SNPs with 1784 markers, while chromosome 4D held only 46 SNPs (Supplementary Fig. S2).

Based on the PCA, the panel clustered in three groups strongly according to their different origins: 66 genotypes from Central-Northern Europe (25.2%), 146 genotypes from Eastern Europe-Western Asia (55.6%), and 42 North-American accessions (16%) (Table S1; Supplementary Fig. S3). Heatmaps kinship matrix with dendrograms confirmed that there are clusters among the accessions based on the used SNPs (Supplementary Fig. S4). The mean r^2^ values for the whole wheat genome decreased with increasing distance between SNPs as Mbp. The average LD decay distance for the whole genome was approximately 2 Mbp (Supplementary Fig. S5).

### Variation in seed size-related traits and correlations

Data analysis revealed extensive phenotypic variation in all studied traits suggesting the suitability of the used panel for association genetic studies. Phenotypic values for each of the six traits were found normally distributed. All variables analyzed were significantly influenced by the years, genotypes, and *G × Y* (environment) interactions (*p < 0.001*) (Table 1). Broad-sense heritability was high ranging from 0.87 (Factor form density) to 0.93 (Kernel length). The main results and summary statistics are indicated in Table S2.

**Table 1:** *P* values and broad sense heritability of Thousand-kernel weight (TKW), Kernel length (KL), Kernel width (KW), Kernel area (KA), Kernel diameter ratio (KDR) and Factor form density (FFD) in an experiment with 261 wheat genotypes evaluated during three years

Trait	Year	Genotype	Genotype×Year	*H*^*2*^
Thousand kernel weight (TKW)	***	***	***	0.90
Kernel length (KL)	***	***	***	0.93
Kernel width (KW)	***	***	***	0.88
Kernel area (KA)	***	***	***	0.90
Kernel diameter ratio (KDR)	***	***	***	0.92
Factor form density (FFD)	***	***	***	0.87

H^2^: broad-sense heritability. *** indicate significance *P* < 0.001

**Fig. 1:**
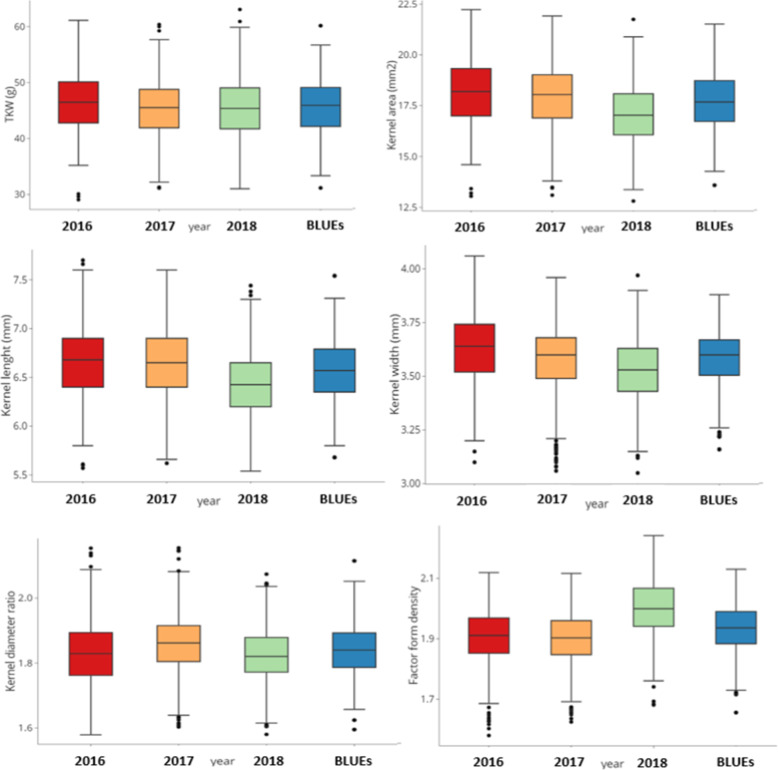
Boxplot for **a** Thousand kernel weight (TKW-g), **b** Kernel area (mm^2^), **c** Kernel width (mm), **d** Kernel length (mm), **e** Kernel diameter ratio and **f** Factor form density in 261 winter wheat genotypes during three years and BLUEs

For TKW, BLUEs values across the three field environments (years) varied from 31.06 to 60.16 g, showing ranges of 29.08 to 61.11 g (2016), 31.15 to 60.37 g (2017), and 31.04 to 63.05 g (2018) as shown in Table S2 and Fig. 1. The KL means ranged from 5.60 to 7.54 mm. Values ranged from 5.57 to 7.70 mm (2016), 5.62 to 7.60 mm (2017), and 5.54 to 7.44 mm in 2018. Kernel width (KW) varied from 3.10 to 4.06 mm (2016); 3.06 to 3.96 mm (2017); 3.05 to 3.97 mm (2018) and 3.14 to 3.88 mm (BLUEs). Variations in KA fluctuated between 13.05–22.23 mm^2^ (2016), 13.10–21.92 mm^2^ (2017), 12.81–21.75 mm^2^ (2018) and 13.26–21.52 mm^2^ (BLUEs). KDR ranged from 1.58 to 2.15 (2016); 1.60 to 2.15 (2017); 1.58 to 2.07 (2018) and 1.59 to 2.11 (BLUEs). BLUEs values for FFD ranged from 1.66 to 2.13. FFD varied between 1.58–2.12 (2016), 1.63–2.12 (2017) and 1.66–2.13 (2018) (Table S2; Fig. 1; Supplementary Fig. S6). Boxplots showing natural phenotypic variation among genotypes across years are indicated in Fig. 1. The evaluated traits showed high heritability (> 0.87) over the 3 years, indicating that most of the traits were stable and largely determined by genetic factors (Table 1 Supplementary Fig. S6).

**Fig. 2:**
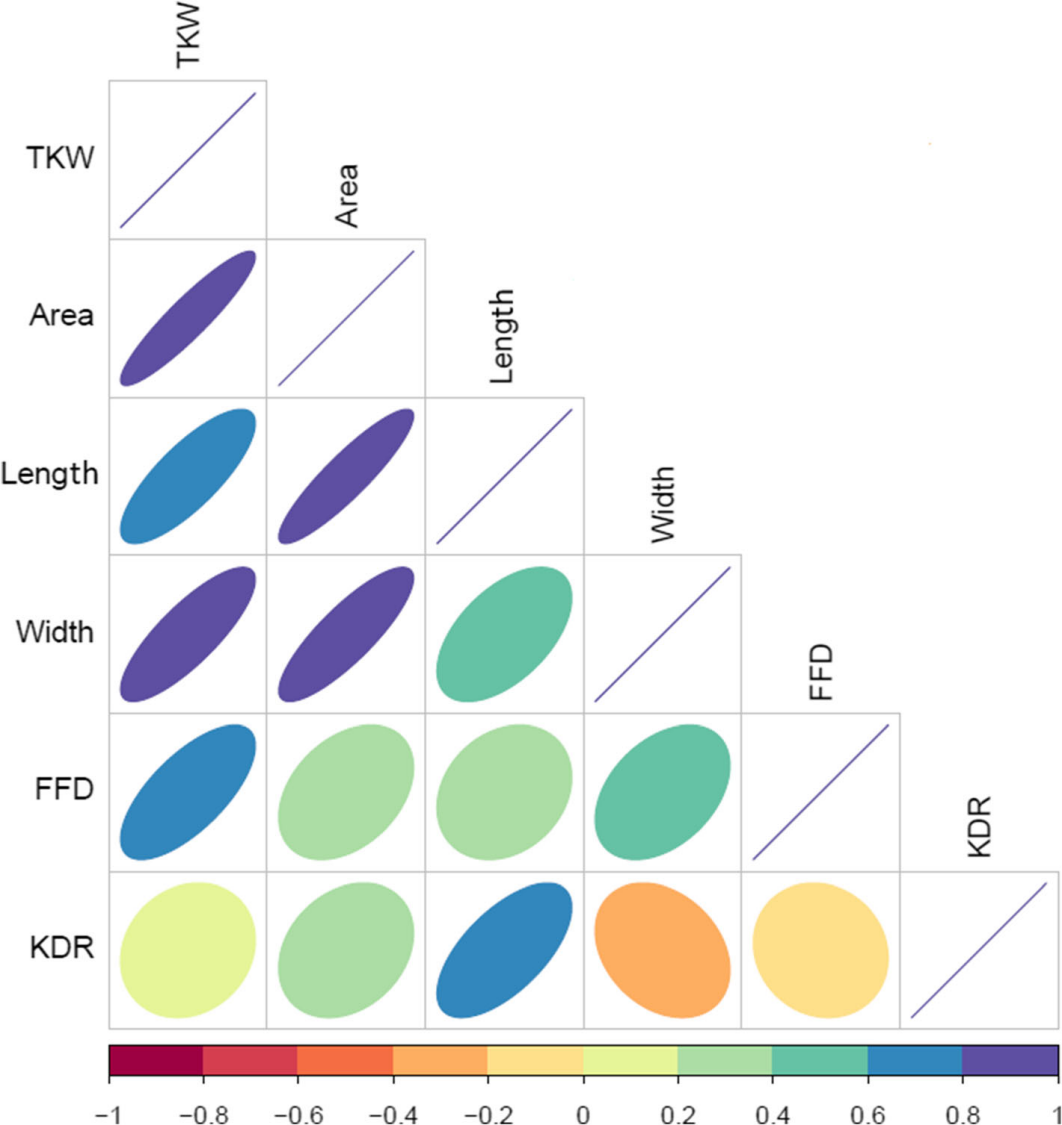
Pearson Correlation Coefficient values in wheat genotypes based on BLUEs value. The degree of significance for all correlations was *p* < 0.001. The color and size of the ellipse reflect the strength of the correlation

TKW showed significant (*P < 0.001*) and positive correlation with KA (0.91), KW (0.82), KL (0.78), FFD (0.68) and a weaker association with KDR (0.13). The KA revealed a strong correlation with KL (0.90), KW (0.84) while weaker associations were detected for KDR (0.24) and FFD (0.33). The KL and KW presented a positive correlation (0.55), while a negative association was reported for KW and KDR (− 0.31) and KDR with FFD (− 0.14) (Fig. 2). The correlation values suggesting that common genetic factors controlling more than two traits are expected to be detected in the current study.

Important differences in these traits were detected when the different origins of the genotypes were contrasted. In this sense, those cultivars from East Europe-Western Asia (e.g., Russia, Kazakhstan, Kirgizstan, Ukraine) presented higher TKW (48.18 g) and KA (18.28 mm^2^) compared to those from Central and Northern Europe (45.36 g; 17.81 mm^2^) (France, Poland, Germany, UK, Romania, Sweden, Finland, etc.) and North America which presented the lowest values (41.41 g; 16.32 mm^2^) (Supplementary Fig. S7).

### Candidate genes underlying grain architecture-related traits in wheat

The highly significant SNP markers which passed the FDR threshold and associated with more than one trait were selected for detecting putative candidate genes. One hundred forty-nine high confidence candidate genes were fallen within the LD interval (2Mbp) (Table 2; Table S4). Some of these genes were found to encode proteins with known functions, however, other genes showed unknown functions. Four SNPs were found to be inside the physical position of four novel candidate genes those were selected according to their annotation and association with several grain architecture traits.

**Table 2:** Distribution of pleiotropic loci associated with two or more grain architecture related traits

Chr	Marker	Trait/Effect/−log_10_(p-value)	Marker Position (bp) and alelles	Candidate gene-Genomic location	Annotation
1B	***wsnp_Ex_c1600_3051075*(IWA2084)**	**TKW (**+ **1.611) 4.20** **KA (+ 0.134) 5.66**	**524,154,547–524,154,747** **T-G**	**TraesCS1B01G303200** **(524153507–524,155,132)**	**Protein of unknown function DUF1677,*O. sativa***
2A	***Excalibur_c12169_1088*** **(IWB22047)**	**TKW (+ 2.176) 6.91** **KA (+ 0.576) 5.76**	**82,350,252–82,350,352** **A-G**	**TraesCS2A01G136800** **(82349905–82,354,821)**	**Heat shock protein binding (Gos)** **Heat shock protein DnaJ, cysteine-rich domain (interpros)**
5B	***RAC875_c9150_2945*(IWB61034)**	**KA (− 0.318) 5.23** **KL (− 0.110) 4.20**	**459,477,406–459,477,506** **C-T**	**TraesCS5B01G274000** **(459476178–459,493,013)**	**P-loop containing nucleoside triphosphate hydrolase (interpros)**
6A	***BobWhite_c5872_589*(IWB4014)**	**TKW (−1.755) 6.34** **KA (−0.516) 5.70**	**602,710,319–602,710,419** **C-T**	**TraesCS6A01G383800** **(602699767–602,711,726)**	**Oxidation-reduction process/MF: oxidoreductase activity/MF: NADP binding/MF: oxidoreductase activity, acting on a sulfur group of donors, NAD(P) as acceptor/BP: cell redox homeostasis/MF: flavin adenine dinucleotide binding/MF: glutathione-disulfide reductase activity/BP: glutathione metabolic process**

*TKW* Thousand-kernel weight, *KL* Kernel length and *KA* Kernel area, *Chr* Chromosome, Position (Physical, bp); −log_10_ (*p*-value (SNP))

The candidate gene *TraesCS1B01G303200* on chromosome 1B (position 524,153,507–524,155,132 bp) was annotated as a protein of unknown function DUF1677 in rice (*O. sativa*). The candidate gene *TraesCS2A01G136800* is located on chromosome 2A (position 82,349,905–82,354,821 bp) and has a role in Heat shock protein DnaJ, cysteine-rich domain. The alleles of SNP Excalibur_c12169_1088 (position 82,350,252–82,350,352 bp) which is located within the interval of the previous candidate gene did not show a significant effect on the associated traits as shown in Fig. 4. On chromosome 5B, SNP RAC875_c9150_2945 (position 459,477,406–459,477,506 bp) is co-located within the candidate gene *TraesCS5B01G274000* (position 459,476,178–459,493,013 bp); the carrying alleles are C and T where allele C affect significantly KA and KL (*p < 0.05*) (Fig. 4). The candidate gene annotates P-loop containing nucleoside triphosphate hydrolase. The last identified candidate gene *TraesCS6A01G383800* is located on chromosome 6A (position 602,699,767–602,711,726 bp) and encodes an oxidoreductase activity, acting on a sulfur group of donors, NAD(P) as acceptor. The SNP BobWhite_c5872_589 located within the gene (position 602,710,319–602,710,419 bp) held C and T alleles, where allele C affected TKW significantly (*p < 0.01*) but had no significant effect on KA.

The expression analysis of candidate genes in different grain tissues showed a wide range of expression for the genes (Fig. 5). In general terms, gene *TraesCS6A01G383800* shows the highest expression in most of the grain tissues (grain, aleurone layer, starchy endosperm and the seed coat, endosperm, grain transfer cells, and whole endosperm) while genes *TraesCS1B01G303200* and *TraesCS2A01G136800* also showed high values. For their part, *TraesCS5B01G274000* gene had very low expression compared with the other three but showed high expression values for embryo proper (Fig. 5).

Interestingly in Chromosome 1A, the SNP TA001286–0611-w showed a significant association with TKW and a weaker association with KA (LOD = 3.18)*.* This region harbors the candidate gene *TraesCS1A01G007200* (position 3,776,265–3,777,399 bp) which annotates Gliadin/LMW glutenin/Bifunctional inhibitor/plant lipid transfer protein/seed storage helical domain (Table S3). The analysis of SNP allele variation in marker TA001286–0611-w (position 3,777,195–3,777,321 bp) showed that allele C has a significant impact on TKW (*p < 0.01*) but had no effect on KA compared to allele T (Data not shown).

### GWAS results

**Fig. 3:**
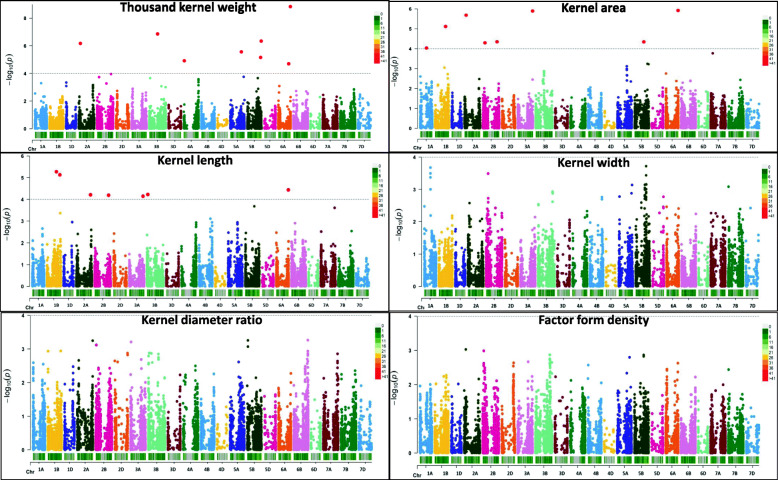
Manhattan plots showing significant marker-trait associations for six traits related to grain architecture in 261 winter wheat genotypes using BLUEs values. Red dots indicate significant markers (*p* < 0.001; −log_10_ > FDR)

In total, 17 MTAs across 9 chromosomes related to grain architecture related-traits were detected above the false discovery rate (FDR) threshold (−log_10_ > FDR). FDR threshold was above than -log_10_ > 4.2 for the studied traits. Therefore, we reduced the number of spurious associations by only considering those SNPs, which exceeded the FDR. Markers were identified on chromosomes 1A (1), 1B (2), 2A (2), 2B (3), 3A (1), 4B (1), 5B (4), 6A (2), 6B (1) (Fig. 3, Supplementary Fig. S8 and Table S3).

For TKW, seven MTAs were identified and present on chromosomes 1A (1), 2A (1), 2B (2), 6A (1) and 6B (1). Four of these markers located on chromosomes 2A, 2B, 6A, and 6B showed highly significant associations (−log_10_ > 5). The most significant marker (−log_10_ = 6.91) was *Excalibur_c12169_1088* located in chromosome 2A and affected this trait by + 2.176 g (R^2^ = 5.2%). The phenotypic variation explained by markers (R^2^) ranged between 3.2 to 5.2% (Supplementary Table S3).

For kernel area, seven MTAs on chromosomes 1B (1), 2A (1), 2B (1), 3A (1), 4B (1), 5B (1) and 6A (1) were reported. Six of these MTAs located on 1B, 2A, 2B, 3A, 5B and 6A showed -log_10_ > 5. For this trait, markers *Tdurum_contig59780_988* located on chromosome 2B (−log_10_ = 6.88, Effect = − 0.408 mm^2^, R^2^ = 2.8%), *Excalibur_c12169_1088* on chromosome 2A (−log_10_ = 5.76, Effect = + 0.576 mm^2^; R^2^ = 1.9%) and *wsnp_Ex_c1600_3051075* on chromosome 1B (−log_10_ = 5.66, Effect = + 0.605 mm^2^, R^2^ = 1.9%) were the most significant ones detected. Marker effects oscillated between 1.9 to 2.6% (Supplementary Table S3).

Three MTAs related to kernel length were detected on chromosome5B (RAC875_c9150_2945, Excalibur_c23709_938 and Kukri_c10530_1013) that showed high effects in these traits. R^2^ reached values of 2.0 to 2.2%.

No MTAs were detected for FFD, KDR and KL when the -log10 > FDR threshold was used. Conversely, several MTAs were reported between the -log10 > 3 and -log10 < FDR range: 5 (KA), 2 (FFD), 19 (KDR), 3 (KA), 1 (TKW), 23 (KL). Information about SNP detected for each trait, their physical position, −log_10_, MAF, effect, related candidate genes, and their functions are shown in Supplementary Table S3.

For all of these markers associated with the studied traits, we detected the closest candidate genes based on the physical position within local blocks of LD. Our results showed that four MTAs across four chromosomes are associated with more than one trait. Those MTAs were detected on 1B (TKW-KA), 2A (TKW-KA), 5B (KA-KL) and 6A (TKW-KA) (Table 2).

## Discussion

### Genotypic variability in grain architecture traits

The identification of factors affecting grain weight and grain architecture is of major importance to accelerate the rate of genetic gain of wheat and increase grain quality. Despite the high number of identified QTL controlling grain morphology in wheat, the implemented studies are scarce or have been carried out partially using bi-parental populations e.g., DH, RILs, and F_2_ populations [14, 15, 21, 26, 27, 34, 37, 38, 44, 46]. This study explores the power of GWAS to identify genomic regions associated with six-grain architecture related-traits in a novel set of 261 winter wheat genotypes, using 17,093 SNP markers in three environments. Our results indicate an extensive phenotypic variation in all traits evaluated across the genotypes. A high heritability (0.87–0.93) and high correlation among environments was observed, indicating the feasibility of this panel for selection of traits related to grain yield and quality improvement [47]. Our results indicate the usefulness of the population used for GWAS studies. In addition, the high diversity of these genotypes can provide more valuable inference compared to bi-parental populations by taking advantage of maximum allelic diversity as was suggested by several authors [48, 49]. Furthermore, the variation on grain architecture-related traits based on population structure presented significant differences among geographical regions showing that those cultivars from Eastern Europe-Western Asia had higher TKW and KA compared to those from Central-Northern Europe and North America.

#### Marker-traits association related to grain architecture

Our analysis detected 17 marker-trait associations related to grain architecture traits across 9 chromosomes, indicates that these are quantitative traits under polygenic control as was reported [26]. These SNPs were passed FDR analysis that provided highly significant true associations (*P*-values ≥ FDR) which can be used in further analysis.

In this study, TKW among 261 winter wheat genotypes showed variability from 31 to 60 g and 7 MTAs related to this trait were detected on chromosomes 1A, 2A, 2B, 5B, 6A, and 6B. Previous studies identified QTL on chromosomes 1A [50], 2A [9, 26, 42, 51], 2B [9, 40, 52], 3A [40, 52–54], 5B [53], 6A [9, 50, 51] and 6B [9, 26, 53]. To the best of our knowledge, the identified MTAs for TKW in this study have not been reported yet and they are potentially novel MTAs responsible for this trait. In addition, candidate genes for TKW have also been identified by several authors [43, 45, 55] but as far as we know, none of them are coincident with the ones reported in this work and its corresponding functions (Table 2).

**Fig. 4:**
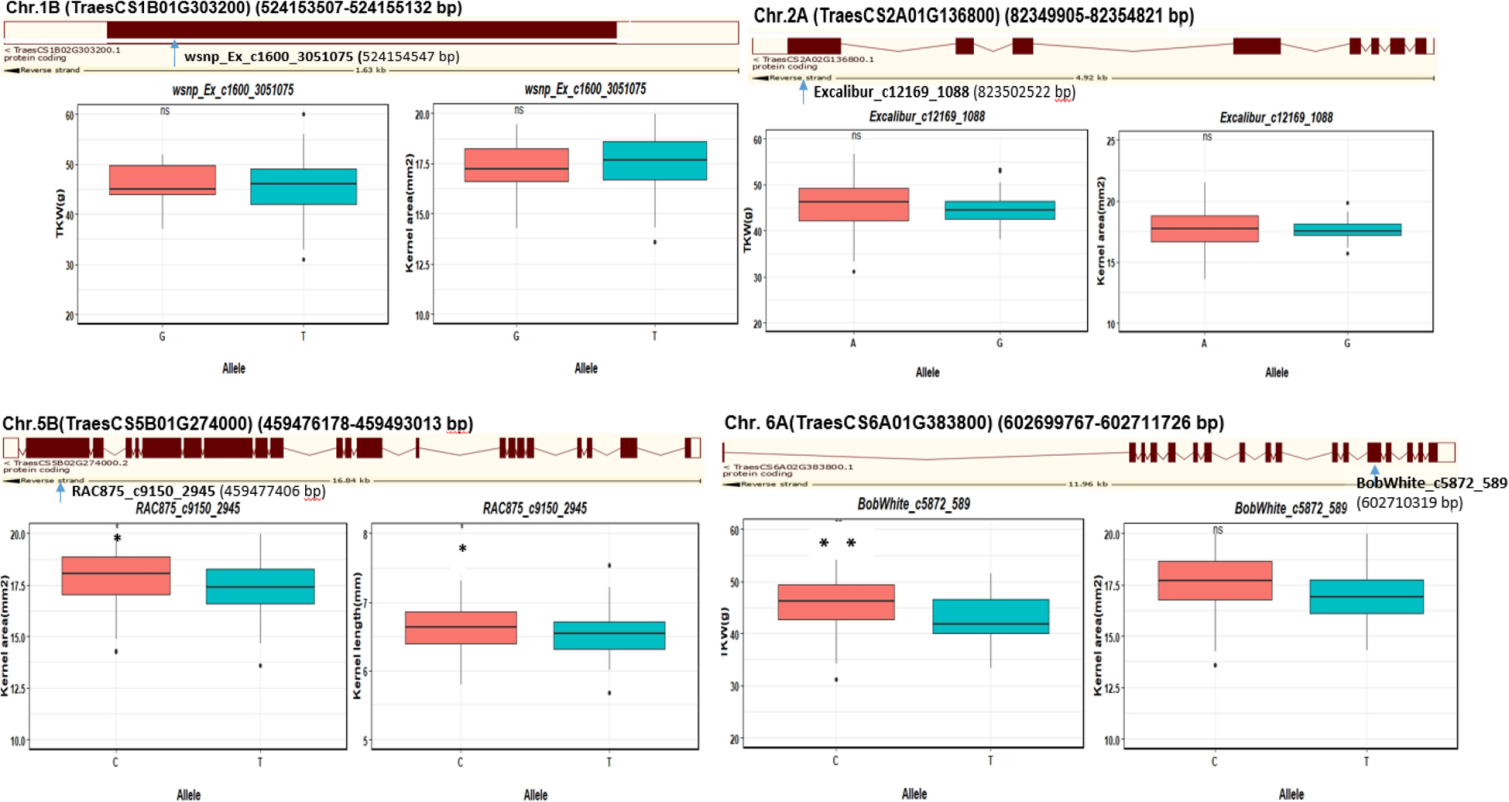
The structure of the candidate genes with the position of the co-located SNPs within the gene and SNP-gene haplotype analysis. The degree of significance is indicated as *p < 0.05; **p < 0.01; ns: not significant

In the same way, extensive genotypic variation and significant G × E interactions were reported for KA, KL, KW, FFD, and KDR. Although previous studies reported QTL associated with KA [26, 51, 56], KL [26, 51, 57–59], KW [59], FFD [58], KDR [20, 58, 60], these markers have been documented in different positions to those found in our work, indicating that the associations reported in our work are novel for these traits. Moreover, four novel MTAs across and related to more than one trait (pleiotropic effects) were detected on chromosomes 1B (TKW-KA), 2A (TKW-KA), 5B (KA-KL), and 6A (TKW-KA) (Table 2; Fig. 4 and Supplementary Fig. S8).

The identification of underlying genes with annotations related to the grain architecture traits provides further reliability for the MTAs identified in the current study. Although high number of candidate genes have been detected, of which four HC candidate genes reported in our work have shown effects in other agronomical traits, none of them have been documented as effective on the size, shape, and weight of the grain and can be considered novel.

In this work, we found that the allelic variation at the locus of the gene *TraesCS1B01G303200* (chromosome 1B) that annotated as Protein of unknown function DUF1677 (*O. sativa*) showed its influence on TKW and KA. Even though Gerard et al. [61] documented an effect of this gene on yield-related traits such as grains per spike, the relationship of this gene with grain architecture has not been previously reported.

The candidate gene *TraesCS2A01G136800* on chromosome 2A has a role in Heat shock protein (HSP) DnaJ, a cysteine-rich domain, which is a major protein in response to stresses ([62], Preprint). The only protein of known function from plants that contains the Cys-rich domain of DnaJ-like proteins is maize BSD2 [63]. The DnaJ-like proteins with other HSP are considered important components in the cytosol and organelles for protein metabolism [64]. Heat stress during the grain filling stage affects the translocation of photosynthates to the grains and the synthesis and deposition of starch [65], resulting in TKW reductions and alterations in the grain morphology. The recent report mention that, the growing season 2017/2018 was the mostest dryness season in IPK since long-ago that influnce spikelet development and abortion in barley [66], that can also explain the effect of heat (high temperture) on the TKW. The synthesis of HSPs is believed to play an important role either in preventing or minimizing the negative effects of high temperature both at the cellular and molecular levels. This gene was related to TKW and KA suggesting its important role in controlling grain weight and architecture that needs further molecular validation. A recent report from Hu et al. [67] documented 3 QTLs associated with heading date (QHD-2A.1), spikelet per spike (QSPS-2A.3), and flag leaf area (QFLA-2A.1) in this region but no effects on grain characteristics have been documented so far.

The candidate gene *TraesCS5B01G274000* on chromosome 5B, annotates P-loop containing nucleoside triphosphate hydrolase (NTPase) which is the most common domain of many nucleotide-binding protein folds. The energy from NTP hydrolysis is typically utilized to induce conformational changes in other molecules, which constitutes the basis of the biological functions of most P-loop NTPases. P-loop NTPases show substantial substrate preference for either ATP or GTP [68]. Although this gene was linked to KA and KL and its role in those important biological functions may derive from this association with grain morphology, its relationship with these treatments has not been previously documented.

**Fig. 5:**
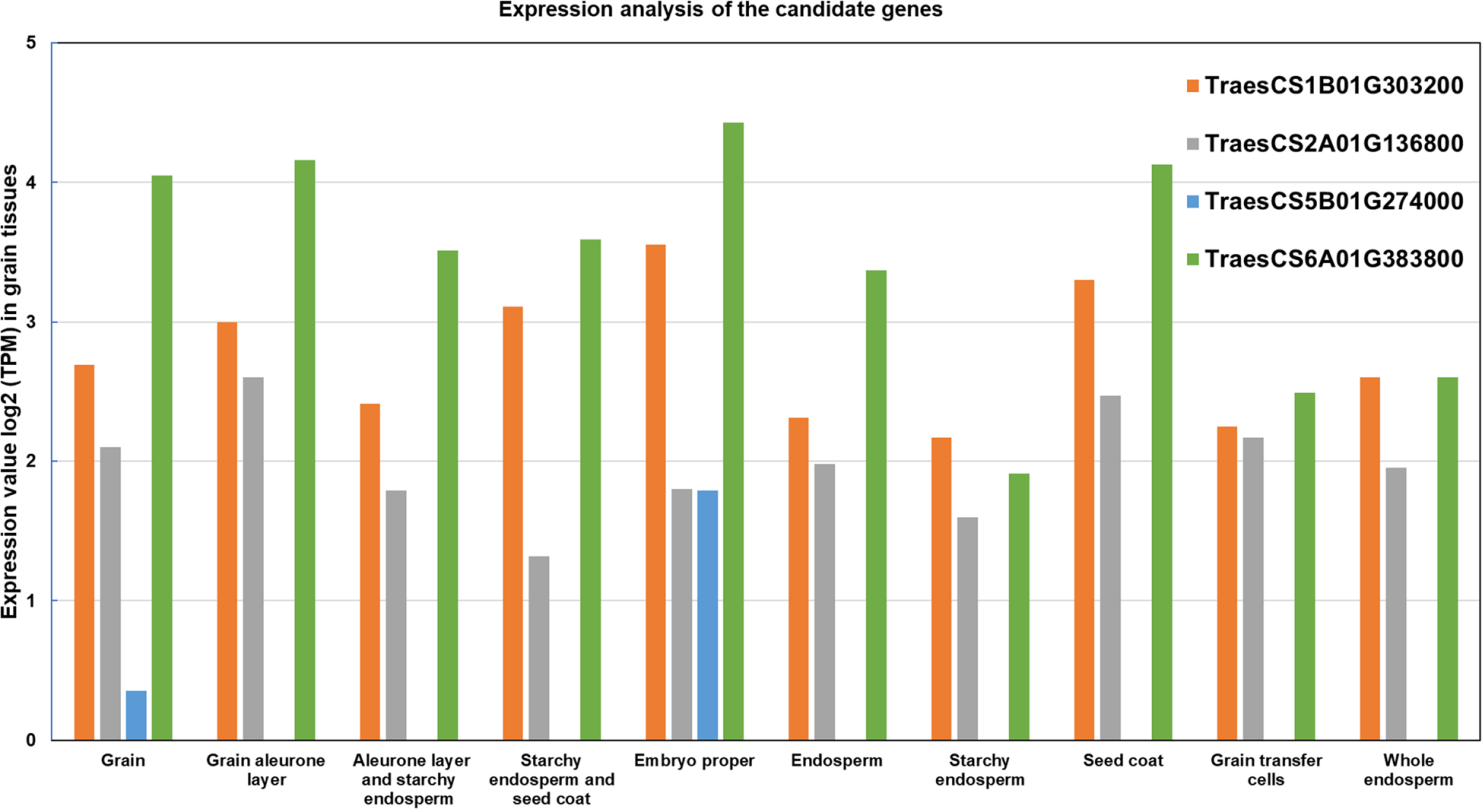
Expression value log_2_ TPM (Transcripts Per Kilobase Million) of candidate genes in different grain tissues

The last identified candidate gene *TraesCS6A01G383800* (chromosome 6A) encodes oxidoreductase activity, acting on a sulfur group of donors, NAD(P) as acceptor. It has been reported that GSH-dependent protein-disulphide oxidoreductase (TPDO) increases the activity in a period of maximum synthesis of storage proteins in wheat grains, which is the third week after anthesis. There is a correlation between TPDO activity in maturing grains and dough extensibility as the enzyme reduces SS bonds in high-molecular-weight polymers [69]. This higher accumulation could result in TKW and KA increases. Effects of this gene were reported on grain quality traits such as Falling Number and root architecture of durum wheat [70] but no association with TKW and KA was reported previously. The high expression of these four candidate genes in different grain related-tissues indicates their important biological role in variables connected to grain architecture-related traits (Fig. 5).

As was previously described, we found that the *TraesCS1A01G007200* gene located on chromosome 1A associated with TKW and with a weaker association with KA has a nutrient reservoir activity molecular function and annotates Gliadin/LMW glutenin//Bifunctional inhibitor/plant lipid transfer protein/seed storage helical domain. It is known that gliadin and glutenin proteins have a major role in grain quality determination [71]. Its role is explained as gliadins and glutenins are the major reserve proteins in wheat [72], forming the gluten and contributing to TKW. Although recent studies reported effects of this gene on gliadin synthesis in durum wheat and heading-anthesis date in hexaploid wheat [73, 74], its effect on traits related to grain architecture and TKW has not been reported so far.

The highly significant SNPs within the candidate genes were analyzed using statistical comparison of alleles at each SNP with the associated traits. This approach have been used recently to explore the alleles for enhancing agronomic traits and abiotic tolerance [12, 75, 76]. The results showed that some SNPs within the candidate genes showed significant differences between alleles of each associated trait considered. Accessions were differentiated according to the alleles which demonstrate their impact on grain architecture-related traits. For example, the allelic variation (C, T) at SNPs BobWhite_c5872_589 underlying the genes *TraesCS6A01G383800,* respectively, showed the importance of allele C in increasing TKW, where most of the accessions carried allele C originated mainly from Central-Northern European countries.

## Conclusions

To our knowledge, our study involves the first GWAS analysis performed by FARM-CPU algorithm to identify several key genomic regions associated with TKW and five other grain architecture traits in a new and wide winter wheat panel consisting of 261 genotypes from 30 countries during three years. Genome-wide association analysis using 90 K SNP array revealed many MTAs including 17 highly associated SNPs across 9 chromosomes of which four multi-traits associated SNPs were found on chromosomes 1B, 2A, 5B, and 6A. We reported four novel candidate genes related to these traits showing high expression values in different grain tissues.

Future studies should deepen the relationships between the function of these genes and their effect on grain architecture. These results will be valuable for identifying regions associated with kernel weight and its dimensions and could be useful for providing further insights for increasing grain quality, milling performance, and grain yield.

## Methods

### Plant materials

In the current study, a winter wheat panel was used comprising 261 accessions including 196 cultivars, 55 breeding lines, and 10 doubled haploids originated from 30 countries worldwide. The seeds of these genotypes were obtained from the Genebank, IPK-Gatersleben, Germany. The accessions were selected based on pre-existing knowledge regarding their performance under different growing conditions during winter time, for instance, high latitude and continental European winter wheat collections as well as Russian and North American cultivars. Furthermore, parts of the core collection of the *Institute of Field and Vegetable Crops* (IFVCNS), Novi Sad, Serbia and parental lines of Western European hybrid breeding programs were also included in this set ([77]; Supplementary Fig. S1; Table S1).

### Grain architecture traits assessments/phenotyping/measurements

Field experiments were carried out at Leibniz Institute of Plant Genetics and Crop Plant Research -IPK- (Gatersleben, Germany) over three consecutive years (2015/2016, 2016/2017 and 2017/2018) with three replicated blocks in 5 m^2^ plots for each accession.

After harvesting, 200 random kernels of each plot (per accession and year) were used to assess kernel size traits including, kernel length (KL; mm), kernel width (KW; mm), kernel area (KA; mm^2^) and TKW (g) using the MARVIN Digital Seed Analyser (MARViTECH GmbH, Germany). In addition, to evaluate the differences in grain density and the deviation of a shape from a cylindrical form, the factor form density (FFD) and kernel diameter ratio (KDR) were calculated according to Giura and Saulescu [13] and Gegas et al. [15] using the following equations:

$$FFD= TKW/\left( KL\ast KW\right)$$

$$KDR= KL/ KW$$

### Statistical analysis

Analysis of variance (ANOVA) for all measured traits was performed and broad-sense heritability (*H*^*2*^) for each trait over years was calculated using the following equation, all these calculations were accomplished using Genstat 19:

$${H}^2=\upsigma {g}^2/\left(\frac{\upsigma {\mathrm{gy}}^2}{\mathrm{y}}+\frac{\upsigma {\mathrm{e}}^2}{\mathrm{ry}}\right)$$

where *σg*^*2*^, *σgy*^*2*^, and *σe*^*2*^ are mean squares for genotype, genotype × year (environment) interaction and residual error, respectively, *y* is the number of years and *r* represent the replicates. Summary statistics across different years were corroborated by mean, minimum, maximum, standard deviation and coefficient of variation using Genstat 19 software. A correlation matrix between traits and boxplots of each trait and differences among geographical regions were performed using MVApp v2.0.

The restricted maximum likelihood (REML) algorithm was applied to calculate the Best Linear Unbiased Estimators (BLUEs) for each trait in each accession across the years by considering the accession as a fixed effect and the environment as a random using Linear and Nonlinear Mixed Effects Models (nlme) package in R [78].

### Genotyping and population structure

The panel was genotyped by TraitGenetics GmbH (http://www.traitgenetics.com) the 90 K iSELECT chip [79]. After removing markers with > 10% missing data and those with the minor allele frequency < 5%, 17,093 SNPs remained (Supplementary Fig. S2). These markers were mapped according to their physical position based on IWGSC RefSeq v1.1 (http://www.wheatgenome.org/, IWGSC RefSeq v1.1) and then used to determine the population structure, linkage disequilibrium (LD) and for GWAS calculation. The genome-wide pairwise estimates of LD were calculated as a squared correlation between pairs of polymorphic SNPs (r^2^) for the whole-genome using GenStat 18 [80]. Finally, LD decay patterns were visualized as the plot for the LD estimated the (r^2^) vs. the distance between pairs of polymorphic SNPs (Mbp) using R-package GGPLOT2 [81]. The average genome-wide LD decay at r^2^ = 0.2 is approximately 2.0 Mbp and we used this window to discover the candidate genes with a reasonable distance as suggested by 3. The genetic relationships among genotypes as the population structure were revealed by principal component analysis (PCA), using Genomic Association and Prediction Integrated Tool (GAPIT 3) package in R [82, 83]. In the present study, the population structure of a diverse panel of 261 wheat genotypes was investigated using the basis of a 90 K SNP chip to specify the number of principal components (PCs) to be included in the GWAS model (Supplementary Fig. S3).

### Genome-wide association study and identifying putative candidate genes

GWAS was performed using BLUEs values in each accession for each trait applying different statistical models through GAPIT in R. FARM-CPU model was applied by considering the random effect model (REM) and the fixed effect model (FEM) iteratively and associated markers as a cofactor that empowered us to avoid any false-negative and control the false positive associations by preventing model overfitting [27, 40, 57, 84–86]. FarmCPU joins the advantages of the mixed linear model and stepwise regression (fixed-effect model) and overcomes their disadvantages by using them iteratively. FarmCPU has higher power and fewer false positives than either MLM or stepwise regression. The detected associations which are passed the threshold of FDR at 0.01 (−log_10_ *P*-values ≥ FDR) were considered as significant marker-trait associations (MTAs). FDR was calculated for each trait separately at the significance level of 0.01 and used in further analyses as recommended by Alqudah et al. [75].

Those highly significant MTAs based on their physical positions were further used to identify the high-confidence (HC) putative candidate genes, based on their physical positions within the LD ± 2 Mbp window, using the reference genome sequence of Chinese Spring by blasting against IWGSC RefSeq annotation v1.1 (http://www.wheatgenome.org/, IWGSC RefSeq v1.1). Because, each block of LD contains high number of candidate genes, we have selected the candidate genes which have SNPs within their physical positions. WheatMine platform (https://urgi.versailles.inra.fr/WheatMine/begin.do) was used to search for the gene ontologies (GO) and InterPro number and description. The underlying genes were further examined for their association with grain architecture traits using previously published literature.

In-silico gene expression analysis in different grain tissues of the multi-traits candidate genes was analyzed using RNA-Seq expression data from Wheat Expression Browser (http://www.wheat-expression.com/).

## Supplementary Information

**Additional file 1: Table S1.** Information about the source, country of origin and biological status of the winter wheat panel. **Table S2.** Summary statistics of Thousand-kernel weight (TKW), Kernel length (KL), Kernel width (KW), Kernel area (KA), Kernel diameter ratio (KDR) and Factor form density (FFD) in an experiment with 261 wheat genotypes evaluated during 3 years. **Table S3.** Significant marker-trait associations and candidate genes for grain architecture traits in 261 winter wheat genotypes.

**Additional file 2: Table S4.** List of candidate genes within the most important four LD blocks.

**Additional file 3: Figure S1.** Origin of the genotypes of the panel. **Figure S2.** Single nucleotide polymorphism (SNP) density on 21 wheat chromosomes. The x-axis shows the interval distance in Mb. **Figure S3.** Screen plot for the first ten principal components (PCs) explains the variation using 90K chip and Population structure based on their origin. **Figure S4.** Kinship heat map of the panel. **Figure S5.** The linkage disequilibrium decay in the wheat population. **Figure S6.** Phenotypic variation and Heritability (*H*^*2*^) for Thousand-kernel weight (TKW), Kernel length (KL), Kernel width (KW), Kernel area (KA), Kernel diameter ratio (KDR) and Factor form density (FFD) using BLUEs values. **Figure S7.** Variation on Thousand-kernel weight (TKW), Kernel length (KL), Kernel width (KW), Kernel area (KA), Kernel diameter ratio (KDR) and Factor form density (FFD) based on the origin of genotypes. C-N Europe (central-northern Europe); E Europe-W Asia (Eastern Europe-Western Asia); N America (North America). **Figure S8.** Overview of significant markers trait associations identified for Thousand-kernel weight (TKW), Kernel length (KL), Kernel width (KW), Kernel area (KA), Kernel diameter ratio (KDR) and Factor form density (FFD) using BLUEs values. Multitraits MTAs are indicated with gray rectangles.

## Data Availability

The datasets supporting the conclusions of this article are included within the article and its supplementary materials published online or are available from the corresponding author on reasonable request. Further information regarding the GenBank accessions including accession name, number and source are available by Babben et al., [7] and Table S1. All accessions used in the current study are publicly available from IPK Gatersleben Genebank.

## Abbreviations

ANOVA: Analysis of variance
BLUE: Best Linear Unbiased Estimators
FFD: Factor form density
FDR: False discovery rate
GWAS: Genome-wide association study
*H*^*2*^: Broad-sense heritability
IWGSC: International Wheat Genome Consortium
KA: Kernal area
KDR: Kernel diameter ratio
KL: Kernel length
KW: Kernel width
LD: Linkage disequilibrium
MAF: Minor allele frequency
PC: Principal component
REML: Residual maximum likelihood
SNP: Single nucleotide polymorphism
TKW: Thousand kernel weight
