# Correction to: Genetic dissection of grain architecture-related traits in a winter wheat population

**DOI:** 10.1186/s12870-021-03216-x

**Published:** 2021-09-29

**Authors:** Matías Schierenbeck, Ahmad M. Alqudah, Ulrike Lohwasser, Rasha A. Tarawneh, María Rosa Simón, Andreas Börner

**Affiliations:** 1 Genebank Department, Leibniz Institute of Plant Genetics and Crop Plant Research (IPK OT Gatersleben), Corrensstr 3, D-06466 Seeland, Germany; 2 Cereals, Faculty of Agricultural Sciences and Forestry, National University of La Plata, La Plata, Argentina; 3 CONICET CCT La Plata.La Plata, Buenos Aires, Argentina; 4 Department of Agroecology, Aarhus University at Flakkebjerg, Forsøgsvej 1, 4200 Slagelse, Denmark

**Correction to: BMC Plant Biol 21, 417 (2021)**

**https://doi.org/10.1186/s12870-021-03183-3**

Following publication of the original article [1], there is an omission in the affiliation of one of the co-authors (María Rosa Simón). The correct affiliation is María Rosa Simón^2,3^.

The original article has been corrected.

## References

[1] Schierenbeck M, Alqudah AM, Lohwasser U (2021). Genetic dissection of grain architecture-related traits in a winter wheat population. BMC Plant Biol.

