# Supplementary material for: Genetic dissection of grain architecture-related traits in a winter wheat population
Source: BMC Plant Biol. 2021 Sep 10;21:417. doi: 10.1186/s12870-021-03183-3 (PMC8431894; doi:10.1186/s12870-021-03183-3)
Supplement: Supplementary file 1 — Additional file 1: Table S1. Information about the source, country of origin and biological status of the winter wheat panel. Table S2. Summary statistics of Thousand-kernel weight (TKW), Kernel length (KL), Kernel width (KW), Kernel area (KA), Kernel diameter ratio (KDR) and Factor form density (FFD) in an experiment with 261 wheat genotypes evaluated during 3 years. Table S3. Significant marker-trait associations and candidate genes for grain architecture traits in 261 winter wheat genotypes. [file 12870_2021_3183_MOESM1_ESM.docx]

**Table S1. Information about the source, country of origin and biological status of the winter wheat panel.**

| Accesion number | Accession name | Source | Country code | Biological Status |
| --- | --- | --- | --- | --- |
| FW1 | Cook | CoreCollection NoviSad via IPK | AUS | cultivar |
| FW2 | Min. Dwarf | CoreCollection NoviSad via IPK | AUS | cultivar |
| FW3 | Triple Dirk B (GK 12) | CoreCollection NoviSad via IPK | AUS | line |
| FW4 | Triple Dirk B (GK 775) | CoreCollection NoviSad via IPK | AUS | line |
| FW5 | Wagrein | n/a | AUT | cultivar |
| FW6 | Suzor'e | NGB | BLR | cultivar |
| FW7 | Antonovka | GSA | BUL | cultivar |
| FW8 | KATYA | HLWWC | BUL | cultivar |
| FW9 | Kristall | GSA | BUL | cultivar |
| FW10 | Neda | GSA | BUL | cultivar |
| FW11 | Rusalka | CoreCollection NoviSad via IPK | BUL | cultivar |
| FW12 | Svilena | GSA | BUL | cultivar |
| FW13 | Zlatica | GSA | BUL | cultivar |
| FW14 | CDC BUTEO | HLWWC | CAN | cultivar |
| FW15 | CDC FALCON | HLWWC | CAN | cultivar |
| FW16 | DH01-25-135*R | HLWWC | CAN | DH line |
| FW17 | DH01-25-199*R | HLWWC | CAN | DH line |
| FW18 | DH01-29-167 | HLWWC | CAN | DH line |
| FW19 | DH01-29-33*R | HLWWC | CAN | DH line |
| FW20 | DH01-32-13 | HLWWC | CAN | DH line |
| FW21 | DH02-15-54 | HLWWC | CAN | DH line |
| FW22 | DH99-39-55-5* | HLWWC | CAN | DH line |
| FW23 | DH02-18-88 | HLWWC | CAN | DH line |
| FW24 | DH01-29-125 | HLWWC | CAN | DH line |
| FW25 | DH99-55-342-4 | HLWWC | CAN | DH line |
| FW26 | PEREGRINE | HLWWC | CAN | line |
| FW27 | S01-249-14*R | HLWWC | CAN | line |
| FW28 | S01-249-8*R | HLWWC | CAN | line |
| FW29 | S01-285-20*R | HLWWC | CAN | line |
| FW30 | S01-285-7*R | HLWWC | CAN | line |
| FW31 | S01-31-12 | HLWWC | CAN | line |
| FW32 | S01-350-6 | HLWWC | CAN | line |
| FW33 | S01-360-1 | HLWWC | CAN | line |
| FW34 | Lambriego Inia | CoreCollection NoviSad via IPK | CHL | cultivar |
| FW35 | Peking 11 | CoreCollection NoviSad via IPK | CHN | cultivar |
| FW36 | Sakura | GSA | CZ | cultivar |
| FW37 | Simila | GSA | CZ | cultivar |
| FW38 | AURA | NGB | FIN | cultivar |
| FW39 | JYVÄ | NGB | FIN | cultivar |
| FW40 | LINNA | NGB | FIN | cultivar |
| FW41 | Vakka | NGB | FIN | cultivar |
| FW42 | APACHE | GSA | FRA | cultivar |
| FW43 | ARLEQUIN | GSA | FRA | cultivar |
| FW44 | AURELE | GSA | FRA | cultivar |
| FW45 | BUENNO | GSA | FRA | cultivar |
| FW46 | Capelle Desprez | CoreCollection NoviSad via IPK | FRA | cultivar |
| FW47 | Durin | CoreCollection NoviSad via IPK | FRA | cultivar |
| FW48 | GSA1 | GSA | FRA | line |
| FW49 | GSA10 | GSA | FRA | line |
| FW50 | GSA3 | GSA | FRA | line |
| FW51 | GSA5 | GSA | FRA | line |
| FW52 | GSA7 | GSA | FRA | line |
| FW53 | MH.98-16 | GSA | FRA | line |
| FW54 | PICARD | GSA | FRA | cultivar |
| FW55 | Avalon | CoreCollection NoviSad via IPK | GBR | cultivar |
| FW56 | Brigant | CoreCollection NoviSad via IPK | GBR | cultivar |
| FW57 | TJB 990-15 | CoreCollection NoviSad via IPK | GBR | line |
| FW58 | AKTEUR | GSA | GER | cultivar |
| FW59 | ARKTIS | GSA | GER | cultivar |
| FW60 | ATHLON | GSA | GER | cultivar |
| FW61 | BRILLIANT | GSA | GER | cultivar |
| FW62 | DISCUS | GSA | GER | cultivar |
| FW63 | FAMULUS | GSA | GER | cultivar |
| FW64 | Flair | GSA | GER | cultivar |
| FW65 | GSA12 | GSA Kursk 2009/10 | GER | line |
| FW66 | JULIUS | GSA | GER | cultivar |
| FW67 | LEIFFER | GSA | GER | cultivar |
| FW68 | MAGISTER | GSA | GER | cultivar |
| FW69 | MATRIX | GSA | GER | cultivar |
| FW70 | Mikon | GSA | GER | cultivar |
| FW71 | Mulan | GSA | GER | cultivar |
| FW72 | PIKO | GSA | GER | cultivar |
| FW73 | Sailor | GSA | GER | cultivar |
| FW74 | SKAGEN | GSA | GER | cultivar |
| FW75 | SW MAXI | GSA | GER | cultivar |
| FW76 | SW TATAROS | GSA | GER | cultivar |
| FW77 | TARSO | GSA | GER | cultivar |
| FW78 | TIGER | GSA | GER | cultivar |
| FW79 | TRANSIT | GSA | GER | cultivar |
| FW80 | Tulsa | GSA | GER | cultivar |
| FW81 | TÜRKIS | GSA | GER | cultivar |
| FW82 | Zentos | GSA | GER | cultivar |
| FW83 | ZOBEL | GSA | GER | cultivar |
| FW84 | Ana | CoreCollection NoviSad via IPK | HRV | cultivar |
| FW85 | ZG 1011 | CoreCollection NoviSad via IPK | HRV | line |
| FW86 | ZG K 3/82 | CoreCollection NoviSad via IPK | HRV | line |
| FW87 | ZG K 238/82 | CoreCollection NoviSad via IPK | HRV | line |
| FW88 | ZG K T 159/82 | CoreCollection NoviSad via IPK | HRV | line |
| FW89 | Bankut 1205 | CoreCollection NoviSad via IPK | HUN | cultivar |
| FW90 | L - 1 | CoreCollection NoviSad via IPK | HUN | line |
| FW91 | Szegedi 768 | CoreCollection NoviSad via IPK | HUN | cultivar |
| FW92 | Suwon 92 | CoreCollection NoviSad via IPK | IND | cultivar |
| FW93 | Acciaio | CoreCollection NoviSad via IPK | ITA | cultivar |
| FW94 | Ai-bian | CoreCollection NoviSad via IPK | JPN | cultivar |
| FW95 | Norin 10 | CoreCollection NoviSad via IPK | JPN | cultivar |
| FW96 | Karabalikskaya Osimaya | GSA | KAZ | cultivar |
| FW97 | KARABALYKSKAYA 101 | HLWWC | KAZ | cultivar |
| FW98 | KARABALYKSKAYA OSTISTAYA | HLWWC | KAZ | cultivar |
| FW99 | KOMSOMOLSKAYA 103 | HLWWC | KAZ | cultivar |
| FW100 | Komsomolskaya 75 | GSA | KAZ | cultivar |
| FW101 | LUTESCENS410H39 | HLWWC | KAZ | cultivar |
| FW102 | LUTESCENS410H48 | HLWWC | KAZ | cultivar |
| FW103 | LUTESCENS410H53 | HLWWC | KAZ | cultivar |
| FW104 | FI-400 | NGB | KG | cultivar |
| FW105 | Pergaja | NGB | LTV | cultivar |
| FW106 | BCD 1302/83 | CoreCollection NoviSad via IPK | MDA | line |
| FW107 | Cajeme 71 | CoreCollection NoviSad via IPK | MEX | cultivar |
| FW108 | SERI82 | HLWWC | MEX | cultivar |
| FW109 | RIDA | NGB | NOR | cultivar |
| FW110 | Almari | GSA | PL | cultivar |
| FW111 | Batuta | GSA | PL | cultivar |
| FW112 | Bogatka | GSA | PL | cultivar |
| FW113 | Finezja | GSA | PL | cultivar |
| FW114 | Kobra plus | GSA | PL | cultivar |
| FW115 | Kohelia | GSA | PL | cultivar |
| FW116 | Korweta | GSA | PL | cultivar |
| FW117 | Legenda | GSA | PL | cultivar |
| FW118 | Muszelka | GSA | PL | cultivar |
| FW119 | Muza | GSA | PL | cultivar |
| FW120 | Naridana | GSA | PL | cultivar |
| FW121 | Narobna | GSA | PL | cultivar |
| FW122 | Roma | GSA | PL | cultivar |
| FW123 | Rywalka | GSA | PL | cultivar |
| FW124 | Slawa | GSA | PL | cultivar |
| FW125 | Smuga | GSA | PL | cultivar |
| FW126 | Sukces | GSA | PL | cultivar |
| FW127 | Tonacja | GSA | PL | cultivar |
| FW128 | Turnia | GSA | PL | cultivar |
| FW129 | Wydma | GSA | PL | cultivar |
| FW130 | Crina | GSA | ROM | cultivar |
| FW131 | 7017 | IC&G SB RAS | RUS | line |
| FW132 | 7109 | IC&G SB RAS | RUS | line |
| FW133 | 7114 | IC&G SB RAS | RUS | line |
| FW134 | 88-85 | HLWWC | RUS | line |
| FW135 | 88-86 | HLWWC | RUS | line |
| FW136 | Al'bina 45 | HLWWC | RUS | cultivar |
| FW137 | Bagrationovskaya | IC&G SB RAS | RUS | cultivar |
| FW138 | Bashkirskaya 10 | VIR | RUS | cultivar |
| FW139 | Bezenchukskaja 380 | GSA | RUS | cultivar |
| FW140 | Bezenchukskaya 380 | VIR | RUS | cultivar |
| FW141 | Bezenchukskaya 616 | VIR | RUS | cultivar |
| FW142 | Bezenchukskaya 790 | VIR | RUS | cultivar |
| FW143 | Bezostaja 1 | CoreCollection NoviSad via IPK | RUS | cultivar |
| FW144 | BEZOSTAYA | HLWWC | RUS | cultivar |
| FW145 | Bezostaya 1 | VIR | RUS | cultivar |
| FW146 | Biruza | GSA | RUS | cultivar |
| FW147 | Biryuza | VIR | RUS | cultivar |
| FW148 | BULAVA | HLWWC | RUS | cultivar |
| FW149 | Doneko | GSA | RUS | cultivar |
| FW150 | Donska polupat. | CoreCollection NoviSad via IPK | RUS | cultivar |
| FW151 | Dzhangal | VIR | RUS | cultivar |
| FW152 | Ermak | GSA | RUS | cultivar |
| FW153 | Ershovskaya 11 | VIR | RUS | cultivar |
| FW154 | Favoritka | GSA | RUS | cultivar |
| FW155 | Filatovka | IC&G SB RAS | RUS | cultivar |
| FW156 | Guberniya | VIR | RUS | cultivar |
| FW157 | Irkutskaya ozimaya | IC&G SB RAS | RUS | cultivar |
| FW158 | Kalach 60 | VIR | RUS | cultivar |
| FW159 | Kazanskaya 285 | VIR | RUS | cultivar |
| FW160 | Kazanskaya 560 | VIR | RUS | cultivar |
| FW161 | Kirgiskaja 16 | GSA | RUS | cultivar |
| FW162 | KP134-3 | HLWWC | RUS | line |
| FW163 | Kuibyshevka | VIR | RUS | cultivar |
| FW164 | Kulundinka | IC&G SB RAS | RUS | cultivar |
| FW165 | Levoberezhnaya 1 | VIR | RUS | cultivar |
| FW166 | Levoberezhnaya 3 | VIR | RUS | cultivar |
| FW167 | M-31*/Jo3088 | IC&G SB RAS | RUS | line |
| FW168 | M-31/Cloud | IC&G SB RAS | RUS | line |
| FW169 | M-31/Flex | IC&G SB RAS | RUS | line |
| FW170 | M-31/Fox | IC&G SB RAS | RUS | line |
| FW171 | M-31/Holley | IC&G SB RAS | RUS | line |
| FW172 | Malakhit | VIR | RUS | cultivar |
| FW173 | Malahit | GSA | RUS | cultivar |
| FW174 | Moskovskaja 56 | GSA | RUS | cultivar |
| FW175 | Moskovskaya 40 | VIR | RUS | cultivar |
| FW176 | Moskovskaya 56 | VIR | RUS | cultivar |
| FW177 | Nemchinovskaya 24 | VIR | RUS | cultivar |
| FW178 | Nemchinovskaya 57 | VIR | RUS | cultivar |
| FW179 | Novosibirskaya 32 | IC&G SB RAS | RUS | cultivar |
| FW180 | Povolzhskaya 86 | VIR | RUS | cultivar |
| FW181 | Rannyaya 12 | VIR | RUS | cultivar |
| FW182 | Resurs | VIR | RUS | cultivar |
| FW183 | Santa | VIR | RUS | cultivar |
| FW184 | Saratovskaya 90 | VIR | RUS | cultivar |
| FW185 | Saratovskaya 17 | VIR | RUS | cultivar |
| FW186 | Severodonetskaya Yubileinaya | VIR | RUS | cultivar |
| FW187 | Skipetr | GSA | RUS | cultivar |
| FW188 | Skorospekla 35 | VIR | RUS | cultivar |
| FW189 | Smuglyanka | VIR | RUS | cultivar |
| FW190 | Svetoch | VIR | RUS | cultivar |
| FW191 | Ul'yanovka | IC&G SB RAS | RUS | cultivar |
| FW192 | UMKA | HLWWC | RUS | cultivar |
| FW193 | Viktoriya 95 | VIR | RUS | cultivar |
| FW194 | Volzhskaya K | VIR | RUS | cultivar |
| FW195 | ZAURALSKAYA OZIMAYA | HLWWC | RUS | cultivar |
| FW196 | Zemka | VIR | RUS | cultivar |
| FW197 | Zvonniza | GSA | RUS | cultivar |
| FW198 | Ivanka | CoreCollection NoviSad via IPK | SRB | cultivar |
| FW199 | L 1/91 | CoreCollection NoviSad via IPK | SRB | line |
| FW200 | Mina | CoreCollection NoviSad via IPK | SRB | cultivar |
| FW201 | Nizija | CoreCollection NoviSad via IPK | SRB | cultivar |
| FW202 | Nov.Crvena | CoreCollection NoviSad via IPK | SRB | cultivar |
| FW203 | Nova banatka | CoreCollection NoviSad via IPK | SRB | cultivar |
| FW204 | NS 22/92 | CoreCollection NoviSad via IPK | SRB | line |
| FW205 | NS 46/90 | CoreCollection NoviSad via IPK | SRB | line |
| FW206 | NS 55-25 | CoreCollection NoviSad via IPK | SRB | line |
| FW207 | NS 602 | CoreCollection NoviSad via IPK | SRB | line |
| FW208 | NS 63-24 | CoreCollection NoviSad via IPK | SRB | line |
| FW209 | NS 33/90 | CoreCollection NoviSad via IPK | SRB | line |
| FW210 | NS 66/92 | CoreCollection NoviSad via IPK | SRB | line |
| FW211 | NS 79/90 | CoreCollection NoviSad via IPK | SRB | line |
| FW212 | PKB Krupna | CoreCollection NoviSad via IPK | SRB | cultivar |
| FW213 | Pobeda | CoreCollection NoviSad via IPK | SRB | cultivar |
| FW214 | Renesansa | CoreCollection NoviSad via IPK | SRB | cultivar |
| FW215 | Sava | CoreCollection NoviSad via IPK | SRB | cultivar |
| FW216 | Slavija | CoreCollection NoviSad via IPK | SRB | cultivar |
| FW217 | Sofija | CoreCollection NoviSad via IPK | SRB | cultivar |
| FW218 | Äring II | NGB | SWE | cultivar |
| FW219 | BANCO | NGB | SWE | cultivar |
| FW220 | BORG | NGB | SWE | cultivar |
| FW221 | FOLKE | NGB | SWE | cultivar |
| FW222 | HOLME | NGB | SWE | cultivar |
| FW223 | KOSACK | NGB | SWE | cultivar |
| FW224 | RENODLAT SAMMETSVETE | NGB | SWE | cultivar |
| FW225 | SAXO | NGB | SWE | cultivar |
| FW226 | STARKE I | NGB | SWE | cultivar |
| FW227 | WALDE | NGB | SWE | cultivar |
| FW228 | Kirija | GSA | UKR | cultivar |
| FW229 | KRUZHINKA/MV IRMA | HLWWC | UKR | cultivar |
| FW230 | Liona | GSA | UKR | cultivar |
| FW231 | Mironovska 808 | CoreCollection NoviSad via IPK | UKR | cultivar |
| FW232 | Mironovskaya 808 | HLWWC | UKR | cultivar |
| FW233 | Odesska 267 | GSA | UKR | cultivar |
| FW234 | Podoljanka | GSA | UKR | cultivar |
| FW235 | Polisska 90 | GSA | UKR | cultivar |
| FW236 | Powaga | GSA | UKR | cultivar |
| FW237 | ST.ERYHTR 1334-07 | HLWWC | UKR | line |
| FW238 | ZOLOTAVA/DAR ZERNOGRADA | HLWWC | UKR | cultivar |
| FW239 | Benni multifloret | CoreCollection NoviSad via IPK | USA | cultivar |
| FW240 | BULK02R2B | HLWWC | USA | line |
| FW241 | Centurk | CoreCollection NoviSad via IPK | USA | cultivar |
| FW242 | Cheyenne | NGB | USA | cultivar |
| FW243 | Ernie | GSA | USA | cultivar |
| FW244 | Florida | CoreCollection NoviSad via IPK | USA | cultivar |
| FW245 | Goldfield | GSA | USA | cultivar |
| FW246 | Hazen | GSA | USA | cultivar |
| FW247 | Helios | CoreCollection NoviSad via IPK | USA | cultivar |
| FW248 | Holly E | CoreCollection NoviSad via IPK | USA | cultivar |
| FW249 | NI98414 | HLWWC | USA | line |
| FW250 | Norstar | GSA | USA | cultivar |
| FW251 | Purd.5392 | CoreCollection NoviSad via IPK | USA | line |
| FW252 | Purd.39120 | CoreCollection NoviSad via IPK | USA | line |
| FW253 | Red Coat | CoreCollection NoviSad via IPK | USA | cultivar |
| FW254 | Ridit | NGB | USA | cultivar |
| FW255 | Roughrider | GSA | USA | cultivar |
| FW256 | SD98444/SD97060 | HLWWC | USA | line |
| FW257 | Vel | CoreCollection NoviSad via IPK | USA | line |
| FW258 | Wesley/SD97049 | HLWWC | USA | line |
| FW259 | n.a.: not available |  |  |  |

**Table S2. Summary statistics of Thousand-kernel weight (TKW), Kernel length (KL), Kernel width (KW),** **Kernel area (KA), Kernel diameter ratio (KDR) and Factor form density (FFD) in an experiment with 261 wheat genotypes evaluated during 3 years**

| Trait | Year | Mean | Min. | Max. | Var. | Median | CV% | *S.D.* |
| --- | --- | --- | --- | --- | --- | --- | --- | --- |
| *Thousand kernel weight (TKW-g)* | **2016** | 46.46 | 29.08 | 61.11 | 27.20 | 46.63 | 11.23 | 5.216 |
|  | **2017** | 45.24 | 31.15 | 60.37 | 27.33 | 45.57 | 11.55 | 5.228 |
|  | **2018** | 45.59 | 31.04 | 63.05 | 26.69 | 45.48 | 11.33 | 5.167 |
|  | **BLUE** | 45.74 | 31.06 | 60.16 | 23.22 | 46.15 | 10.54 | 4.819 |
| *Kernel length (KL-mm)* | **2016** | 6.659 | 5.565 | 7.70 | 0.133 | 6.696 | 5.484 | 0.365 |
|  | **2017** | 6.644 | 5.617 | 7.603 | 0.126 | 6.653 | 5.347 | 0.355 |
|  | **2018** | 6.438 | 5.542 | 7.443 | 0.115 | 6.431 | 5.269 | 0.339 |
|  | **BLUE** | 6.579 | 5.604 | 7.537 | 0.166 | 6.572 | 5.175 | 0.340 |
| *Kernel width (KW-mm)* | **2016** | 3.641 | 3.099 | 4.056 | 0.025 | 3.647 | 4.319 | 0.157 |
|  | **2017** | 3.572 | 3.063 | 3.964 | 0.028 | 3.599 | 4.689 | 0.167 |
|  | **2018** | 3.528 | 3.051 | 3.968 | 0.020 | 3.536 | 4.030 | 0.142 |
|  | **BLUE** | 3.579 | 3.137 | 3.878 | 0.020 | 3.60 | 3.946 | 0.141 |
| *Kernel area (KA-mm^2^)* | **2016** | 18.17 | 13.05 | 22.23 | 2.373 | 18.20 | 8.480 | 1.541 |
|  | **2017** | 17.94 | 13.10 | 21.92 | 2.537 | 18.05 | 8.88 | 1.593 |
|  | **2018** | 17.13 | 12.81 | 21.75 | 2.104 | 17.08 | 8.469 | 1.450 |
|  | **BLUE** | 17.73 | 13.26 | 21.52 | 2.049 | 17.71 | 8.072 | 1.432 |
| *Kernel diameter ratio (KDR)* | **2016** | 1.830 | 1.579 | 2.154 | 0.008 | 1.829 | 5.085 | 0.093 |
|  | **2017** | 1.862 | 1.603 | 2.155 | 0.008 | 1.863 | 4.834 | 0.090 |
|  | **2018** | 1.826 | 1.580 | 2.074 | 0.006 | 1.823 | 4.484 | 0.082 |
|  | **BLUE** | 1.839 | 1.595 | 2.115 | 0.007 | 1.842 | 4.549 | 0.083 |
| *Factor form density (FFD)* | **2016** | 1.911 | 1.580 | 2.119 | 0.007 | 1.913 | 4.571 | 0.087 |
|  | **2017** | 1.900 | 1.625 | 2.116 | 0.007 | 1.904 | 4.528 | 0.086 |
|  | **2018** | 2.000 | 1.681 | 2.241 | 0.008 | 2.001 | 4.551 | 0.091 |
|  | **BLUE** | 1.937 | 1.656 | 2.126 | 0.006 | 1.937 | 4.050 | 0.078 |

Min.: Minimum; Max.: Maximum; Var.: Variance; %CV: coefficient of variation; s.d.: standard deviation

**Table S3. Significant marker-trait associations and candidate genes for grain architecture traits in 261 winter wheat genotypes**

| Trait | SNP | Chr | Position (bp) | -log_10_ (SNP) | Allels | R^2^(%) | Effect (±) | Candidate gene  and position (bp) | Annotation (Interpros) |
| --- | --- | --- | --- | --- | --- | --- | --- | --- | --- |
| KA | **TA001286-0611-w** | 1A | 3777258 | 3.07 | C-T | 1.9 | -0.471 | **TraesCS1A01G007200**  **(3777195-3777321)** | Bifunctional inhibitor/plant lipid transfer protein/seed storage helical domain- Gliadin/LMW glutenin |
| KA | **wsnp_Ex_c1600_3051075** | 1B | 524154647 | 5.66 | T-G | 1.9 | 0.605 | **TraesCS1B01G303200**  **(524153507-524155132)** | Protein of unknown function (DUF1668) |
|  | **Excalibur_c12169_1088** | 2A | 82350302 | 5.76 | A-G | 1.9 | 0.576 | **TraesCS2A01G136800**  **(82349905-82354821)** | Heat shock protein DnaJ, cysteine-rich domain |
|  | **Tdurum_contig59780_988** | 2B | 98365755 | 6.88 | A-C | 2.8 | -0.408 | **TraesCS2B01G132000**  **(98361044-98366963)** |  |
|  | **RFL_Contig5153_958** | 3A | 731085765 | 5.19 | A-G | 2.1 | 0.314 | **TraesCS3A01G511700**  **(731083694-731089749)** | MAC/Perforin domain |
|  | **Kukri_c18722_56** | 4B | 649603299 | 4.59 | A-G | 2.3 | 0.324 | **TraesCS4B01G359300**  **(649602163-649607404)** | WD domain, G-beta repeat |
|  | **RAC875_c9150_2945** | 5B | 459477456 | 5.23 | C-T | 2.6 | -0.318 | **TraesCS5B01G274000**  **(459476178-459493013)** | AAA domain//AAA domain |
|  | **BobWhite_c5872_589** | 6A | 602710369 | 5.70 | C-T | 2.3 | -0.517 | **TraesCS6A01G383800**  **(602699767-602711726)** | Pyridine nucleotide-disulphide oxidoreductase, dimerisation domain/Pyridine nucleotide-disulphide oxidoreductase, class I, active site/FAD/NAD-linked reductase, dimerisation domain/FAD/NAD(P)-binding domain/Glutathione-disulphide reductase |
| KL | **RAC875_c9150_2945** | 5B | 459477456 | 4.20 | C-T | 2.2 | -0.111 | **TraesCS5B01G274000**  **(459476178-459493013)** | P-loop containing nucleoside triphosphate hydrolase |
|  | **Excalibur_c23709_938** | 5B | 460677120 | 4.01 | A-G | 2.0 | -0.109 | **TraesCS5B01G274800**  **(460676432-460679526)** | F-box domain |
|  | **Kukri_c10530_1013** | 5B | 460678316 | 4.01 | A-G | 2.0 | -0.109 | **TraesCS5B01G274800**  **(460676432-460679526)** | F-box domain |
| TKW | **TA001286-0611-w** | 1A | 3777258 | 4.46 | C-T | 3.2 | -1.675 | **TraesCS1A01G007200**  **(3776265-3777399)** | Gliadin/LMW glutenin//Bifunctional inhibitor/plant lipid transfer protein/seed storage helical domain |
|  | **wsnp_Ex_c1600_3051075** | 1B | 524154647 | 4.19 | T-G | 3.2 | 1.611 | **TraesCS1B01G303200**  **(524153507-524155132)** | Protein of unknown function DUF1677, Oryza sativa |
|  | **Excalibur_c12169_1088** | 2A | 82350302 | 6.91 | A-G | 5.2 | 2.176 | **TraesCS2A01G136800**  **(82349905-82354821)** | Heat shock protein DnaJ, cysteine-rich domain |
|  | **Tdurum_contig59780_988** | 2B | 98365755 | 5.59 | A-C | 4.3 | -1.268 | **TraesCS2B01G132000**  **(98361044-98366963)** |  |
|  | **RAC875_c22463_494** | 2B | 767170221 | 4.20 | A-G | 3.3 | -0.907 | **TraesCS2B01G579100**  **(767168671-767172075)** | FAD/NAD(P)-binding domain//Flavin amine oxidase//Amine oxidase |
|  | **BobWhite_c5872_589** | 6A | 602710369 | 6.34 | C-T | 3.7 | -1.755 | **TraesCS6A01G383800**  **(602699767-602711726)** | Pyridine nucleotide-disulphide oxidoreductase, dimerisation domain/Pyridine nucleotide-disulphide oxidoreductase, class I, active site//FAD/NAD-linked reductase, dimerisation domain//FAD/NAD(P)-binding domain//Glutathione-disulphide reductase |
|  | **BobWhite_c47040_185** | 6B | 719509188 | 5.02 | T-C | 3.3 | 1.032 | **TraesCS6B01G471500**  **(719508577-719512868)** |  |

**Thousand-kernel weight (TKW), Kernel length (KL) andKernel area (KA). Chr: Chromosome; Position (Physical, pb); -log_10_ (p-value (SNP)).**
