# Supplementary material for: Genetic dissection of grain architecture-related traits in a winter wheat population
Source: BMC Plant Biol. 2021 Sep 10;21:417. doi: 10.1186/s12870-021-03183-3 (PMC8431894; doi:10.1186/s12870-021-03183-3)
Supplement: Supplementary file 3 — Additional file 3: Figure S1. Origin of the genotypes of the panel. Figure S2. Single nucleotide polymorphism (SNP) density on 21 wheat chromosomes. The x-axis shows the interval distance in Mb. Figure S3. Screen plot for the first ten principal components (PCs) explains the variation using 90K chip and Population structure based on their origin. Figure S4. Kinship heat map of the panel. Figure S5. The linkage disequilibrium decay in the wheat population. Figure S6. Phenotypic variation and Heritability (H2) for Thousand-kernel weight (TKW), Kernel length (KL), Kernel width (KW), Kernel area (KA), Kernel diameter ratio (KDR) and Factor form density (FFD) using BLUEs values. Figure S7. Variation on Thousand-kernel weight (TKW), Kernel length (KL), Kernel width (KW), Kernel area (KA), Kernel diameter ratio (KDR) and Factor form density (FFD) based on the origin of genotypes. C-N Europe (central-northern Europe); E Europe-W Asia (Eastern Europe-Western Asia); N America (North America). Figure S8. Overview of significant markers trait associations identified for Thousand-kernel weight (TKW), Kernel length (KL), Kernel width (KW), Kernel area (KA), Kernel diameter ratio (KDR) and Factor form density (FFD) using BLUEs values. Multitraits MTAs are indicated with gray rectangles. [file 12870_2021_3183_MOESM3_ESM.pptx]

## Slide 1
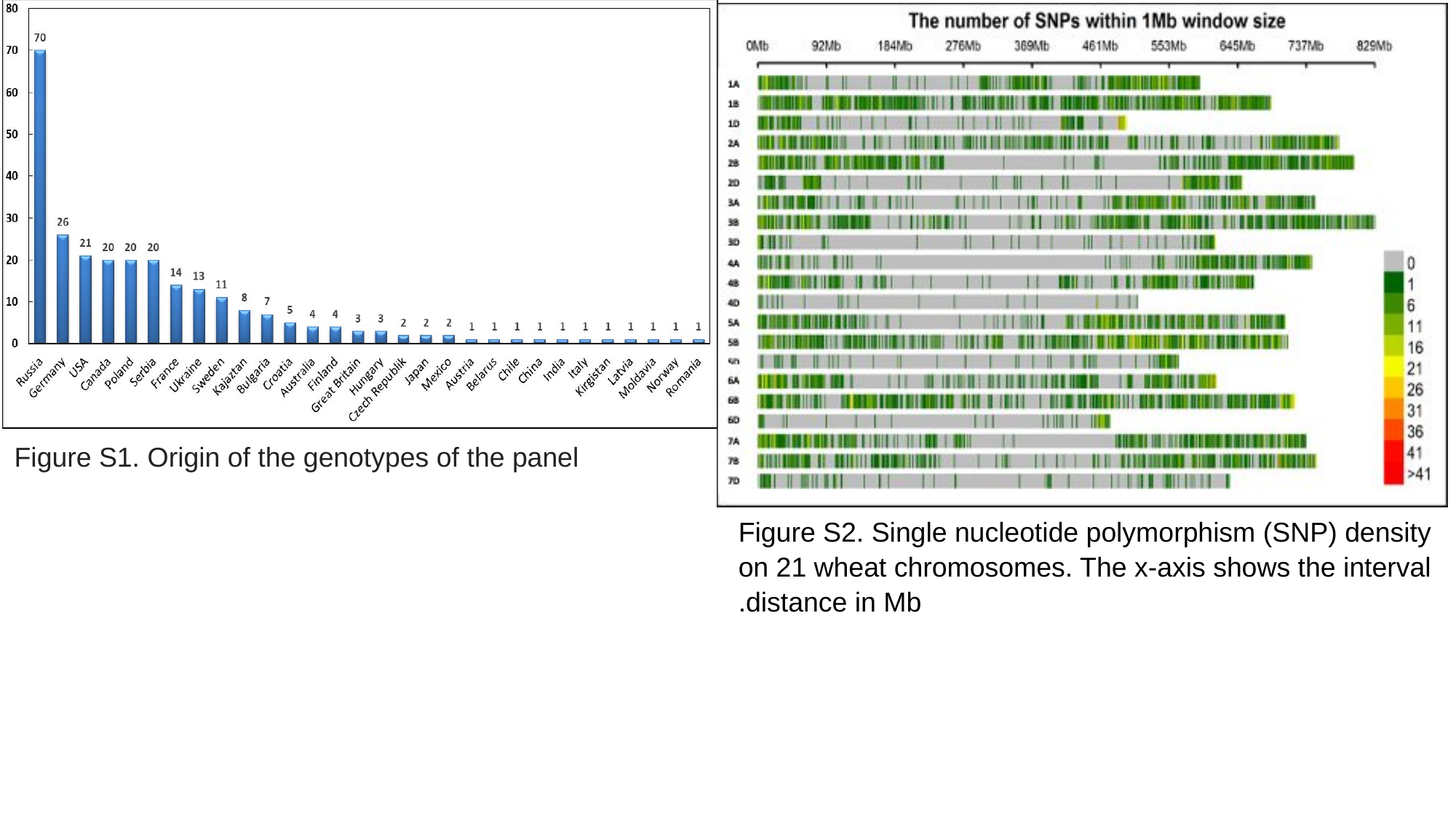

Figure S1. Origin of the genotypes of the panel
Figure S2. Single nucleotide polymorphism (SNP) density on 21 wheat chromosomes. The x-axis shows the interval distance in Mb.

## Slide 2
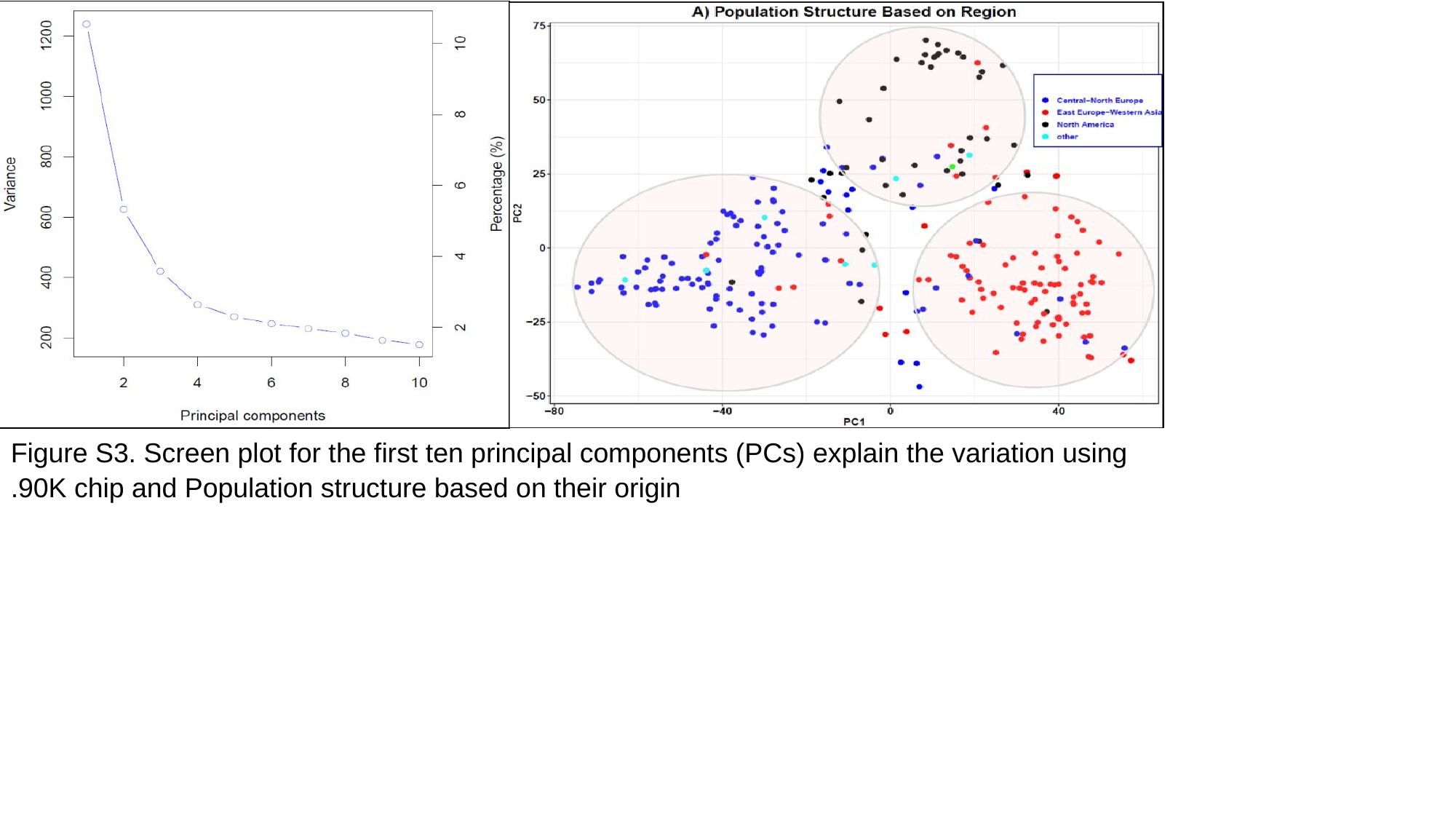

Figure S3. Screen plot for the first ten principal components (PCs) explain the variation using 90K chip and Population structure based on their origin.

## Slide 3
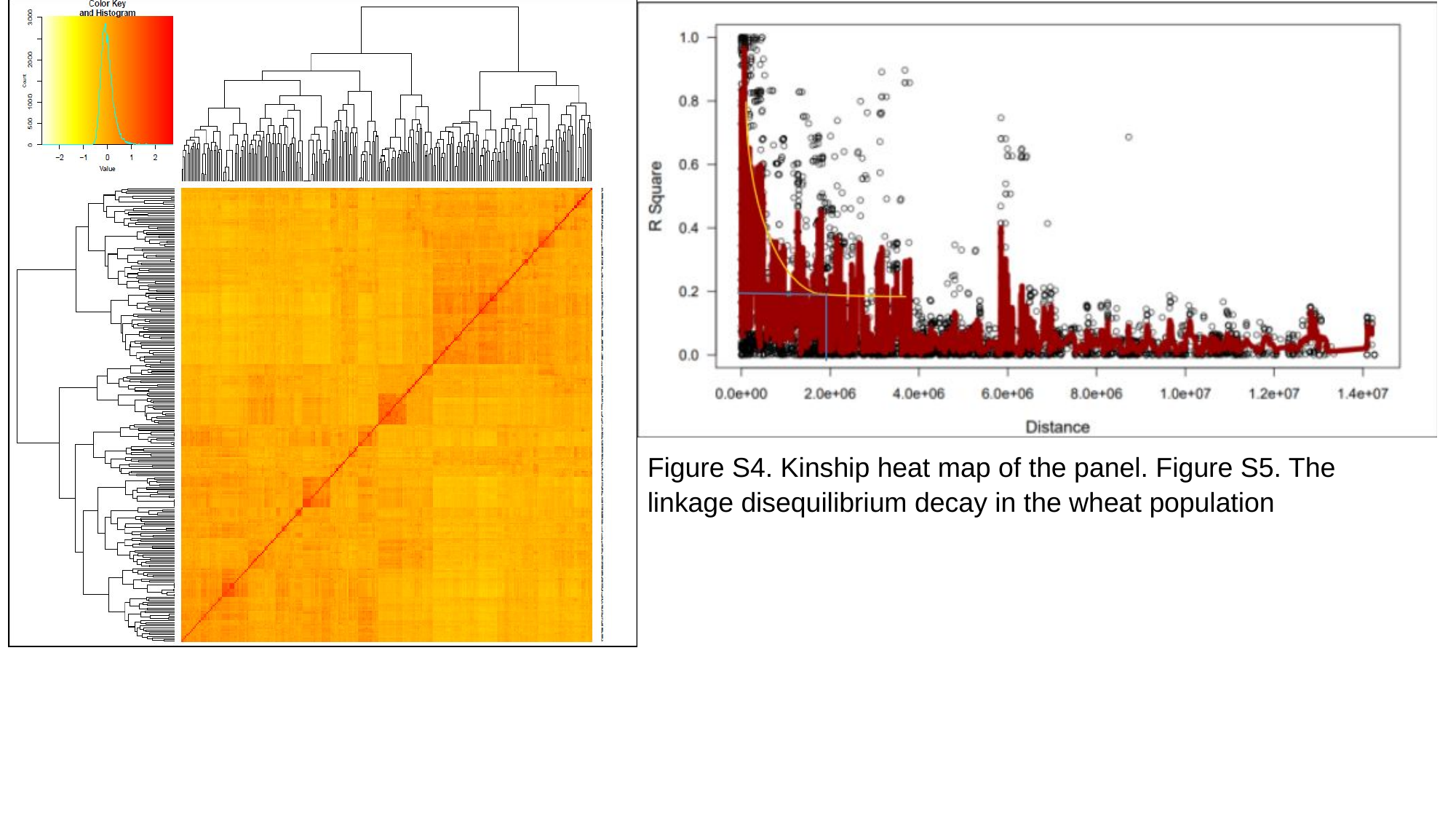

Figure S4. Kinship heat map of the panel. Figure S5. The linkage disequilibrium decay in the wheat population

## Slide 4
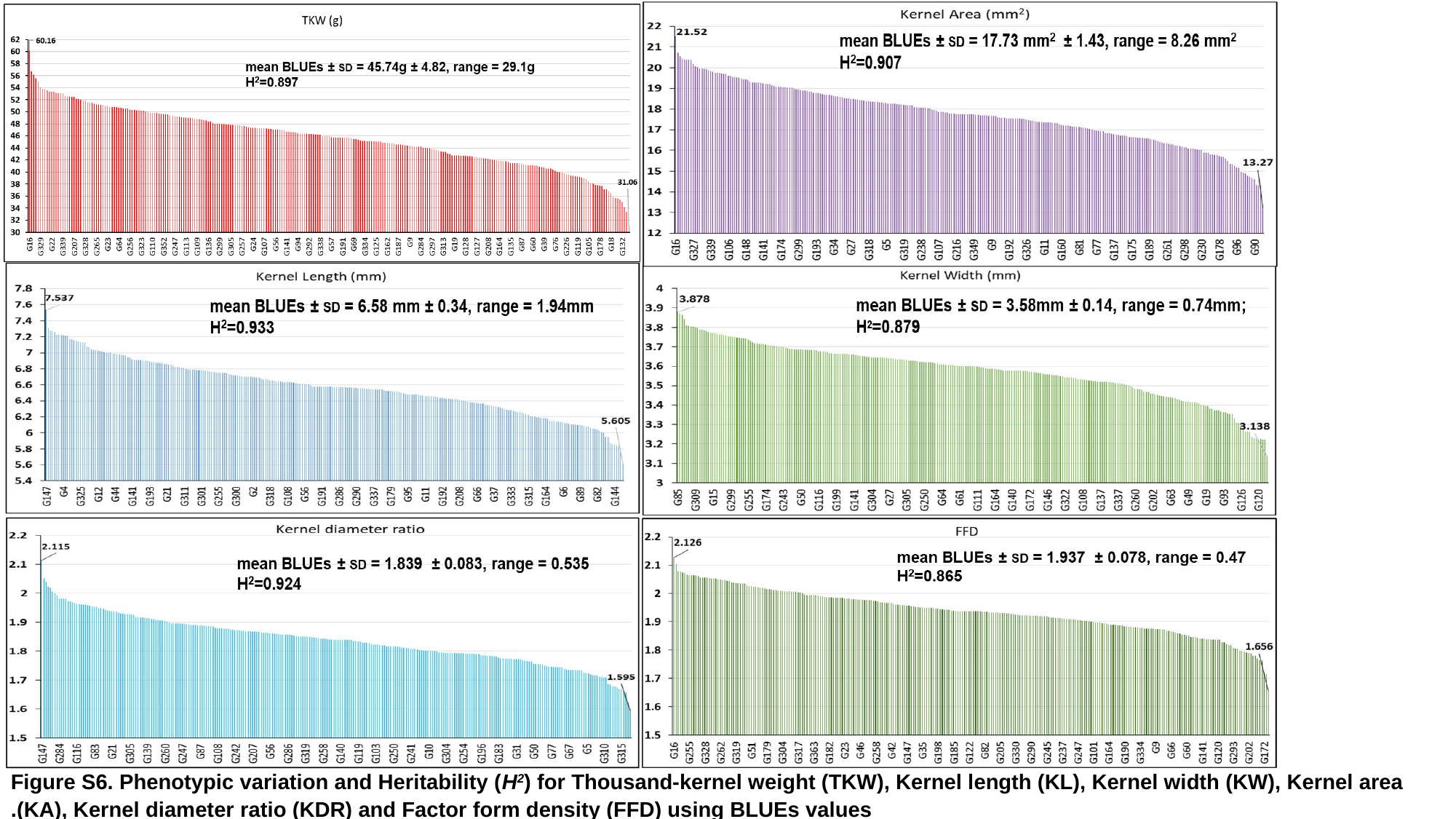

Figure S6. Phenotypic variation and Heritability (H2) for Thousand-kernel weight (TKW), Kernel length (KL), Kernel width (KW), Kernel area (KA), Kernel diameter ratio (KDR) and Factor form density (FFD) using BLUEs values.

## Slide 5
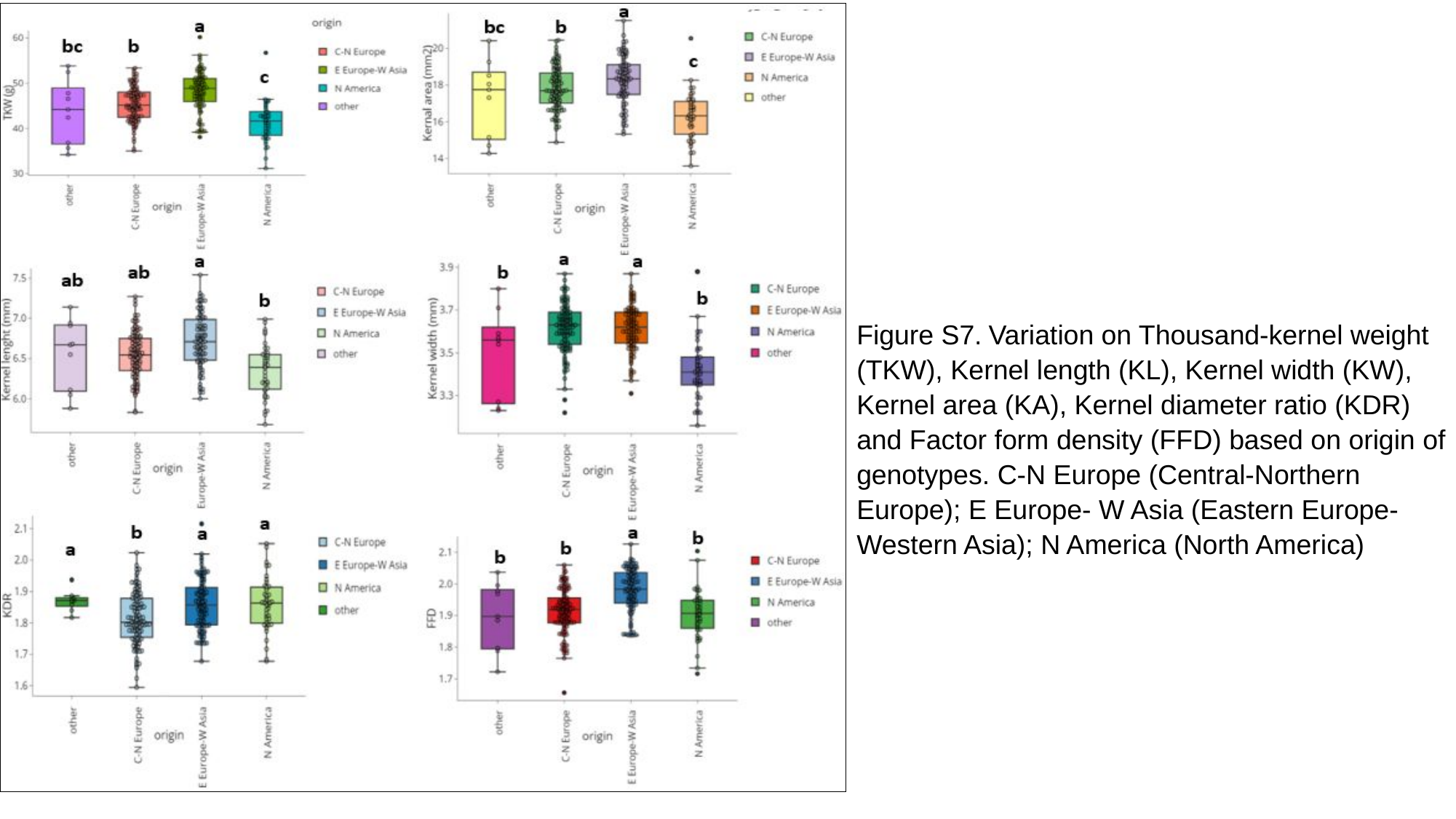

Figure S7. Variation on Thousand-kernel weight (TKW), Kernel length (KL), Kernel width (KW), Kernel area (KA), Kernel diameter ratio (KDR) and Factor form density (FFD) based on origin of genotypes. C-N Europe (Central-Northern Europe); E Europe- W Asia (Eastern Europe-Western Asia); N America (North America)

## Slide 6
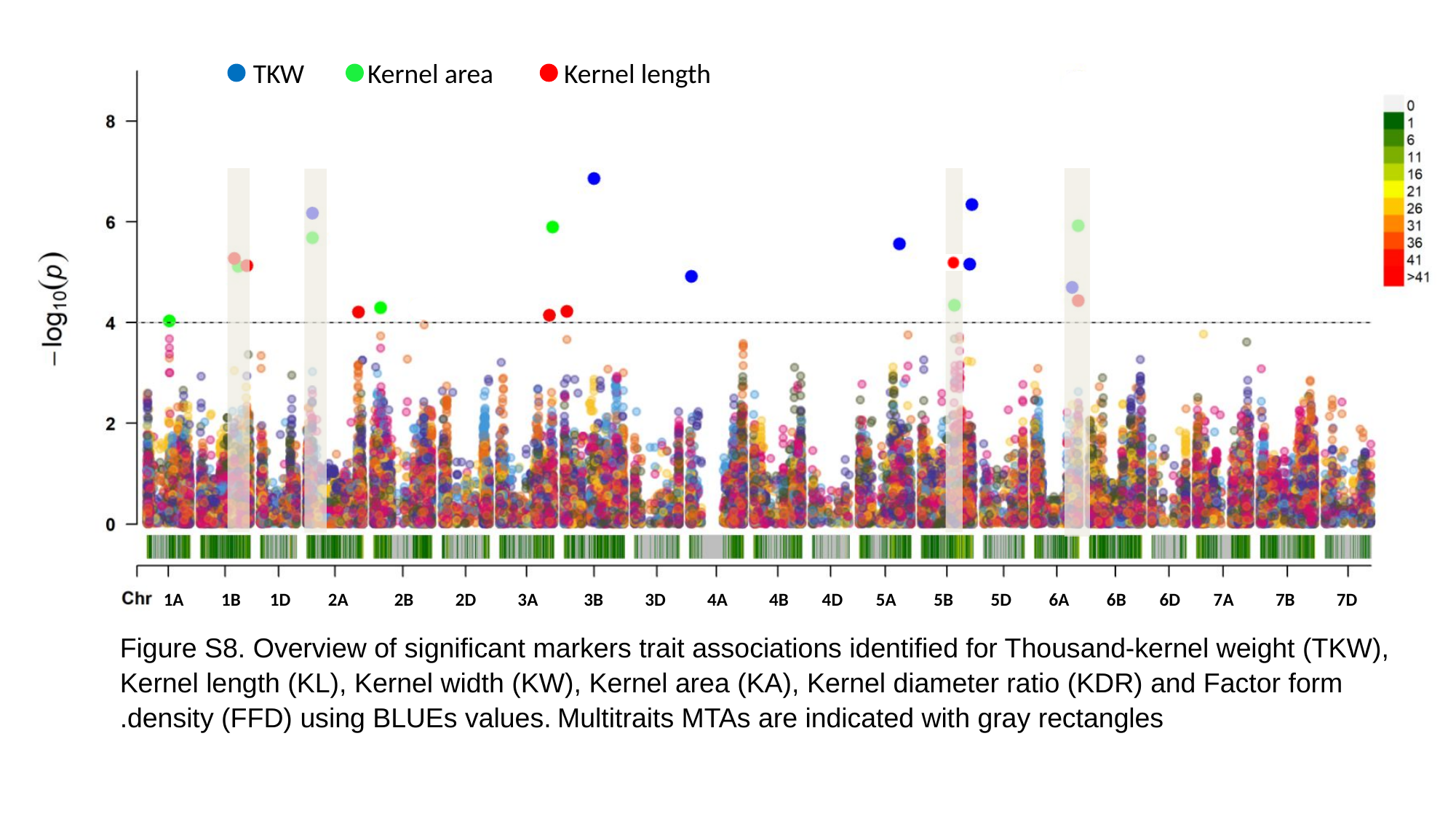

TKW
Kernel area
Kernel length
TKW
Kernel area
Kernel length
1A 1B 1D 2A 2B 2D 3A 3B 3D 4A 4B 4D 5A 5B 5D 6A 6B 6D 7A 7B 7D
1A 1B 1D 2A 2B 2D 3A 3B 3D 4A 4B 4D 5A 5B 5D 6A 6B 6D 7A 7B 7D
Figure S8. Overview of significant markers trait associations identified for Thousand-kernel weight (TKW), Kernel length (KL), Kernel width (KW), Kernel area (KA), Kernel diameter ratio (KDR) and Factor form density (FFD) using BLUEs values. Multitraits MTAs are indicated with gray rectangles.
